# Optogenetic perturbation and bioluminescence imaging to analyze cell-to-cell transfer of oscillatory information

**DOI:** 10.1101/gad.294546.116

**Published:** 2017-03-01

**Authors:** Akihiro Isomura, Fumiko Ogushi, Hiroshi Kori, Ryoichiro Kageyama

**Affiliations:** 1 Institute for Frontier Life and Medical Sciences, Kyoto University, Kyoto 606-8507, Japan;; 2 Japan Science and Technology Agency, PRESTO (Precursory Research for Embryonic Science and Technology), Saitama 332-0012, Japan;; 3 Department of Information Sciences, Ochanomizu University, Tokyo 112-8610, Japan;; 4 Institute for Integrated Cell-Material Sciences (World Premier International research Center [WPI]-iCeMS), Kyoto University, Kyoto 606-8501, Japan;; 5 Graduate School of Medicine, Kyoto University, Kyoto 606-8501, Japan;; 6 Graduate School of Biostudies, Kyoto University, Kyoto 606-8501, Japan

**Keywords:** optogenetics, oscillatory expression, synchronization, frequency tuning, phase shifting, Notch signaling, live imaging

## Abstract

Cells communicate with each other to coordinate their gene activities at the population level through signaling pathways. It has been shown that many gene activities are oscillatory and that the frequency and phase of oscillatory gene expression encode various types of information. However, whether or how such oscillatory information is transmitted from cell to cell remains unknown. Here, we developed an integrated approach that combines optogenetic perturbations and single-cell bioluminescence imaging to visualize and reconstitute synchronized oscillatory gene expression in signal-sending and signal-receiving processes. We found that intracellular and intercellular periodic inputs of Notch signaling entrain intrinsic oscillations by frequency tuning and phase shifting at the single-cell level. In this way, the oscillation dynamics are transmitted through Notch signaling, thereby synchronizing the population of oscillators. Thus, this approach enabled us to control and monitor dynamic cell-to-cell transfer of oscillatory information to coordinate gene expression patterns at the population level.

## Abstract (precis)

Isomura et al. developed an integrated approach that combines optogenetic perturbations and single-cell bioluminescence imaging to visualize and reconstitute synchronized oscillatory gene expression in signal-sending and signal-receiving processes.

Cells communicate with each other by sending and receiving various types of information through signaling pathways. In many cases, the responses of receiving cells depend on the levels of ligands from sending cells, and therefore the amplitude of ligands is part of the information transmitted from cell to cell. However, recent studies revealed that many gene activities are oscillatory and that not only the amplitude but also the frequency and phase convey information for cellular activities (Levine et al. 2013; Purvis and Lahav 2013; Isomura and Kageyama 2014). For example, NF-κB, which exhibits nuclear–cytoplasmic shuttling upon activation of TNFα signaling, induces the downstream gene expression differently according to the shuttling frequencies (Ashall et al. 2009). Similarly, phosphorylated ERK (pERK) levels are pulsatile upon activation of EGF signaling, and the frequencies, rather than the amplitudes, of pERK oscillations are important for the cell proliferation rate (Albeck et al. 2013; Aoki et al. 2013). Interestingly, such ERK activation propagates like waves in the wounded skin, suggesting that waves of ERK activation coordinate cell proliferation for wound healing (Hiratsuka et al. 2015). These results suggest that the frequencies of oscillatory gene activities carry various information for cellular events, but how such oscillatory information is propagated from cell to cell remains unknown.

It has been shown that pulsatile ligand expression is involved in cell-to-cell transfer for coordinating cellular activities at the population level. One such example is the chemoattractant signal cAMP, which is produced in a pulsatile manner and released to neighboring cells during the aggregation stage of *Dictyostelium* (Tomchik and Devreotes 1981; Gregor et al. 2010). These pulses are relayed and propagated like traveling waves, which regulate the collective cell movement (Tyson and Murray 1989). Another example is Delta-like1 (Dll1), a ligand for Notch signaling, which is expressed in an oscillatory manner in the mouse presomitic mesoderm (PSM) (Maruhashi et al. 2005; Bone et al. 2014; Shimojo et al. 2016). Dll1 oscillation is also propagated like traveling waves through PSM cells, and each cycle leads to the formation of a pair of somites. These results raise the possibility that pulsatile ligand expression is involved in cell-to-cell transfer of oscillatory information.

Dll1 oscillation is driven by the Notch effectors Hes1 and Hes7, whose expression oscillates robustly and synchronously between neighboring PSM cells (Jouve et al. 2000; Bessho et al. 2001). However, when PSM cells were dissociated, both *Hes1* and *Hes7* oscillations became unstable and noisy, suggesting that cell-to-cell communication plays a role in robust and synchronized oscillations (Maroto et al. 2005; Masamizu et al. 2006). Indeed, when these dissociated PSM cells were aggregated, they resumed robust and synchronized oscillations within 5–6 h even though they were derived from several embryos (Tsiairis and Aulehla 2016). The exact mechanism for such robust synchronization remains to be determined, but previous analyses using genetic perturbations or inhibitor application revealed that the Notch signaling pathway is required for synchronized oscillation (Jiang et al. 2000; Horikawa et al. 2006; Riedel-Kruse et al. 2007; Delaune et al. 2012; Shimojo et al. 2016; Tsiairis and Aulehla 2016). However, it is not known whether and how single-cell genetic oscillators transmit and decode dynamic information through Notch signaling and whether Dll1 oscillation is sufficient to convey such information from cell to cell for synchronization.

The key to analyzing this issue is the ability to deliver oscillatory gene expression with various frequencies at multiple nodes and monitor the responses in real time at the single-cell resolution. To this end, we developed an optogenetic approach based on the LightOn/GAVPO system (Wang et al. 2012) combined with a method of monitoring gene expression by live imaging of bioluminescence reporters at the single-cell resolution. By using this approach, we found that periodic inputs of Notch signaling entrain intrinsic oscillations by frequency tuning and phase shifting, revealing the mechanism for cell-to-cell transfer of the oscillatory information.

## Results

### Optogenetic perturbations

**Figure 1.**
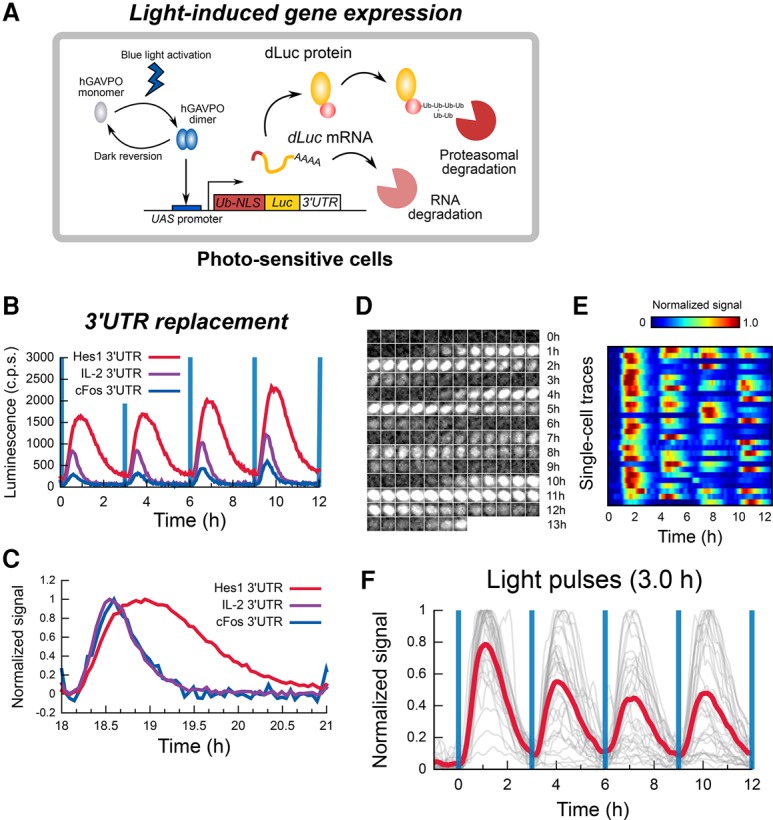
Optogenetic perturbation system. (*A*) Schematic of the light-inducible gene expression system based on hGAVPO. Blue-light illumination activates hGAVPO, which induces downstream gene expression under the control of the UAS promoter. Here, the effects of 3′ untranslated region (UTR) sequences on the optogenetic input were examined by using the ubiquitinated nuclear localization signal (Ub-NLS)-luciferase (Luc) construct. (*B*) The effects of 3′ UTR sequences on light-induced gene expression patterns. (c.p.s.) Counts per second. (*C*) The effects of 3′ UTR sequences on light-induced gene expression patterns in *B* were normalized. (*D*–*F*) Single-cell bioluminescence imaging of oscillatory gene expression patterns induced by the construct containing the *Hes1* 3′ UTR with a 3-h period of blue-light illumination. (*D*) Snapshots for a single cell imaged for 13.5 h. (*E*) A heat map of single-cell traces. Each line represents a cell. (*F*) Population (red) and single-cell (gray) time series.

To deliver oscillatory gene expression with various dynamics, we first developed an optogenetic perturbation system using the codon-optimized GAVPO (hGAVPO), which consists of *Neurospora crassa* photoreceptor Vivid, the Gal4 DNA-binding domain, and the p65 activation domain (Wang et al. 2012; Imayoshi et al. 2013). Upon blue-light illumination, hGAVPO forms a dimer through Vivid, binds to the UAS sequences via a dimer form of the Gal4 DNA-binding domain, and activates the downstream gene expression via the p65 activation domain (Fig. 1A). In a dark condition, hGAVPO dissociates back to a monomer, and the downstream gene expression is switched off (Fig. 1A).

We compared the effects of 3′ untranslated region (UTR) sequences on the optogenetic input by using the ubiquitinated nuclear localization signal (Ub-NLS)-luciferase (Luc) construct under the control of the UAS promoter (Fig. 1A; Supplemental Fig. S1A), a reporter that is unstable like many signaling factors such as Hes1 and Dll1 proteins (Hirata et al. 2002; Shimojo et al. 2016). The 3′ UTR sequences of *Hes1*, *IL-2*, and *cFos* mRNAs, which have short half-lives, were able to generate periodic expression on an ultradian time scale (as short as 1.83-h periodicity) by the hGAVPO-based and UAS promoter-based optogenetic system (Fig. 1B,C), whereas the 3′ UTR of the SV40 late gene, which has a longer half-life, was not (Supplemental Fig. S1B). Among those generating ultradian oscillations, the *Hes1* 3′ UTR exhibited the highest amplitude and the longest duration of on phase (Fig. 1B,C). Thus, in the present study, we used the *Hes1* 3′ UTR to deliver oscillatory gene expression, which was able to generate robust oscillation at the single-cell level (Fig. 1D–F; Supplemental Movie S1).

### Integrated approach for controlling and visualizing oscillatory gene expression

**Figure 2.**
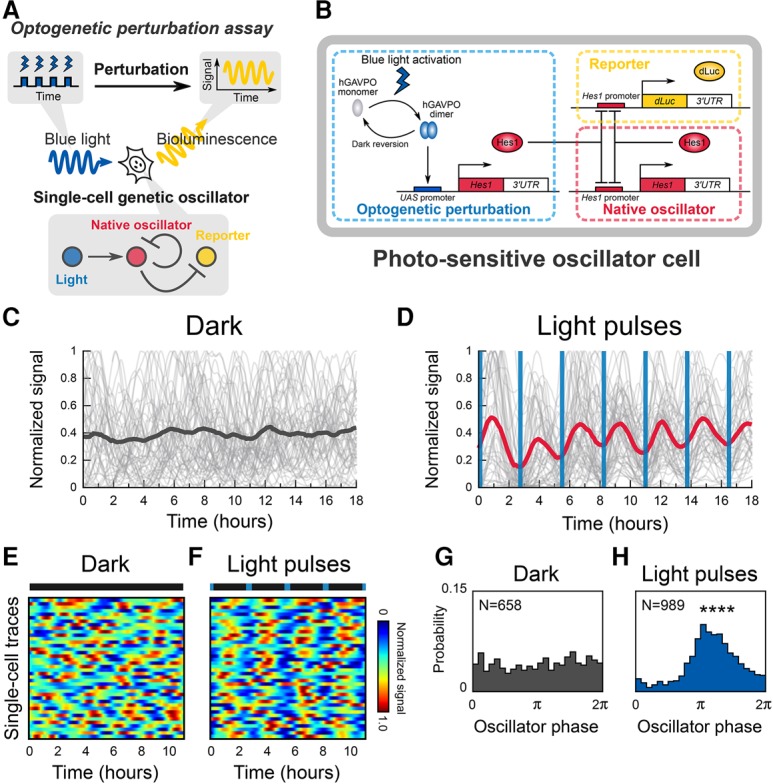
An integrated approach for visualizing and controlling a natural genetic oscillator. (*A*) Schematic of the single-cell optogenetic perturbation experiments in a cell-autonomous system. (*B*) Schematic of the genetic network comprising a bioluminescent reporter (dLuc) and an optogenetic perturbation module using hGAVPO, which are connected to a native oscillator module of the Hes1 negative feedback loop. (*C*) Single-cell time series (gray lines) and a trace of the population average of luminescent signals (black line) in a dark condition. (*D*) Single-cell time series (gray lines) and a trace of the population average of luminescent signals (red line) in the presence of 2.75-h period blue-light pulses. Blue vertical lines represent the timing of illumination with 2-min duration. (*E*,*F*) Heat maps represent single-cell traces in the dark (*E*) and with light pulses (*F*). (*F*) Blue markers in a bar indicate the timing of light illumination. (*G*,*H*) Phase distribution of individual cells in a dark condition (*G*) or in the presence of 2.75-h period blue-light pulses (*H*). *P* = 0.015 for a dark condition; (****) *P* < 0.0001 (Rayleigh test).

To control and visualize gene expression dynamics, we next developed an integrated method that combines the above-described optogenetic perturbation system with single-cell bioluminescence imaging of downstream gene expression (Fig. 2A). We applied this approach to the Notch effector gene *Hes1*, which encodes a basic helix–loop–helix transcriptional repressor that suppresses its own transcription by binding to its own promoter (Hirata et al. 2002). *Hes1* expression oscillates with 2- to 3-h periodicity in many cell types, and this oscillation depends on negative feedback of Hes1 on *Hes1* transcription (Hirata et al. 2002; Shimojo et al. 2008). While this oscillation is unstable and noisy when cells are dissociated or Notch signaling is inhibited, it is robust and stable in intact tissues such as the PSM, suggesting that Notch-mediated cell-to-cell interactions play an important role in coordinating gene expression (Masamizu et al. 2006). Thus, we decided to use our method to analyze the entrainment and synchronization processes of *Hes1* oscillations.

By using this optogenetic perturbation system, we asked how genetic oscillators decode dynamic inputs at the single-cell level. To address this question, we generated photosensitive oscillator cells (derived from C2C12 murine myoblast cells) in which endogenous *Hes1* expression oscillates by negative feedback (Fig. 2B, native oscillator; Hirata et al. 2002). These engineered cells carried two exogenous genetic modules for perturbation and visualization (Supplemental Fig. S2B). For perturbation, we introduced the above-described hGAVPO system, which can induce *Hes1* expression upon blue-light illumination (Fig. 2B, optogenetic perturbation). Feeder cells expressing Dll1 in a constitutive manner were cocultured to induce basal levels of *Hes1* expression (Supplemental Fig. S2A–C). The light-induced Hes1 protein (HA-Hes1) levels were comparable with the endogenous Hes1 protein levels (Supplemental Fig. S3A,B). For visualization of the endogenous *Hes1* expression, we used destabilized luciferase (*dLuc*) driven by the *Hes1* promoter (Fig. 2B, reporter; Masamizu et al. 2006).

We made time-lapse movies of *Hes1* reporter-driven luminescence images and tracked single-cell traces of the endogenous *Hes1* expression from these movies (Supplemental Movies S2, S3). Under dark conditions, the population average of the endogenous *Hes1* expression displayed a quenched pattern (Fig. 2C, black line). In contrast, cyclic blue-light illumination induced an oscillatory pattern with the same periodicity as the illumination, indicating population-level synchrony of the oscillators (Fig. 2D, red line; Supplemental Fig. S3C). Single-cell traces of luminescence signals were pulsatile in both dark and periodic illumination conditions (Fig. 2C–F, gray lines; Supplemental Fig. S2D), suggesting that the dampened population trace under dark conditions is not due to the absence of oscillation in individual cells but due to nonsynchronized oscillations.

We next performed single-cell phase analysis of the oscillators. Time series of luminescence movies of *Hes1* oscillations in individual cells were smoothened and subjected to Hilbert transformation to obtain the phase information (Supplemental Fig. S4A–E). This analysis showed that the phase distribution of single-cell oscillators is random and nondirectional under dark conditions (Fig. 2G; Supplemental Fig. S4F; Supplemental Movie S2). In contrast, the phase distribution was directional after light exposure (Fig. 2H; Supplemental Fig. S4G; Supplemental Movie S3). Together, these data showed that the cyclic optogenetic perturbation elicits synchronized oscillation at the population level, which is indicative of the entrainment of single-cell genetic oscillators by periodic external forcing (Pikovsky et al. 2001).

### Entrainment of the endogenous *Hes1* oscillation by intracellular Hes1 inputs

**Figure 3.**
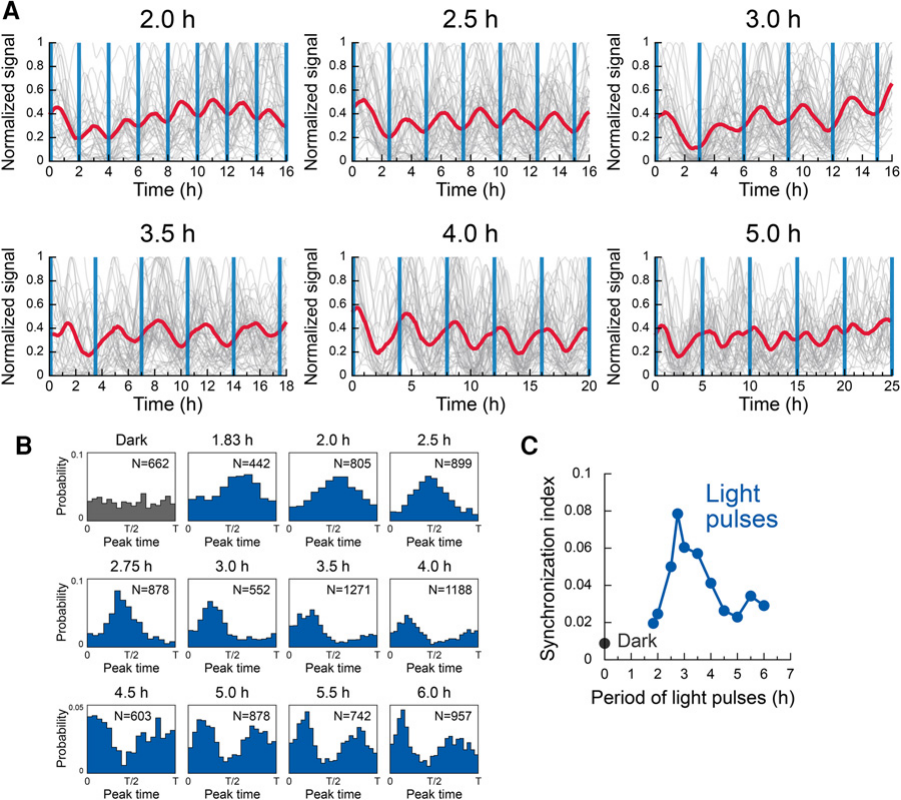
Entrainment of a population of genetic oscillators by periodic optogenetic perturbation. (*A*) Single-cell traces (gray) and traces of population average (red) in the presence of periodic perturbation at periodicities ranging from 2.0 to 5.0 h. Blue vertical lines represent timings of illumination of a 2-min duration. (*B*) Peak time distributions under the dark condition or perturbation at periodicities ranging from 1.83 to 6.0 h. *N* represents the number of analyzed peak events. (*C*) Synchronization index calculated by the entropy-based method.

Next, we investigated the entrainment process by applying various periods of blue-light illumination. At the population level, oscillators were entrained to optogenetic pulses with a range of periods from 2 to 5 h (Fig. 3A). Under these conditions, the periods of the population dynamics corresponded to those of blue-light illumination. Interestingly, when the stimuli with a 5-h period were applied, bimodal responses appeared within each stimulation cycle, indicating that optogenetic pulses induced population synchrony in a resonant-like manner (Fig. 3A).

To characterize these resonant-like responses at the single-cell level, we examined the probability distributions of peak timings. The peak maxima in a time series of luminescence signals were detected, and the times after stimulation were mapped (Supplemental Fig. S5A). Under dark conditions, the probability distribution of peak timings was statistically uniform (*P* = 0.72, Rayleigh test) (Fig. 3B, dark), suggesting an asynchronous state in a population of individual oscillators. In contrast, pulsatile light illumination with 1.83- to 6.0-h periodicity induced strongly directional distributions (*P* < 0.0001, Rayleigh test) (Fig. 3B), indicating a synchronous state of oscillators. At periodicity of 5 h or longer, bimodal structures appeared, suggesting resonant-like responses by the oscillators at the single-cell level (Fig. 3B).

We next sought to compute a synchronization index to quantify the degree of synchronization using an entropy-based measure (Tass et al. 1998). This synchronization index showed that the best condition for entrainment was 2.75-h periodicity (Fig. 3C), which is similar to the period of free-running oscillators under dark conditions (2.57 h ± 0.96 h) (see the Supplemental Material). Furthermore, we found a second peak of 5.5-h periodicity (Fig. 3C), suggesting an entrainment of the order 2:1; i.e., two pulses within one oscillatory cycle of external stimulus. Another measure of synchronization efficiency based on angular statistics (also known as the Kuramoto order parameter) showed that maxima appeared at 2.75- and 5.5-h periods (Supplemental Fig. S5B). These resonant-like behaviors of self-sustained oscillators in the presence of periodic external forcing are known as Arnold tongue regions (Pikovsky et al. 2001; Mondragón-Palomino et al. 2011; Kellogg and Tay 2015). Taken together, these results suggest that the *Hes1* oscillator persists in maintaining cyclic dynamics with an intrinsic period and that periodic signaling inputs that match the intrinsic period preferentially synchronize individual oscillators.

### Stochastic phase model for dynamic responses of the Hes1 oscillators

**Figure 4.**
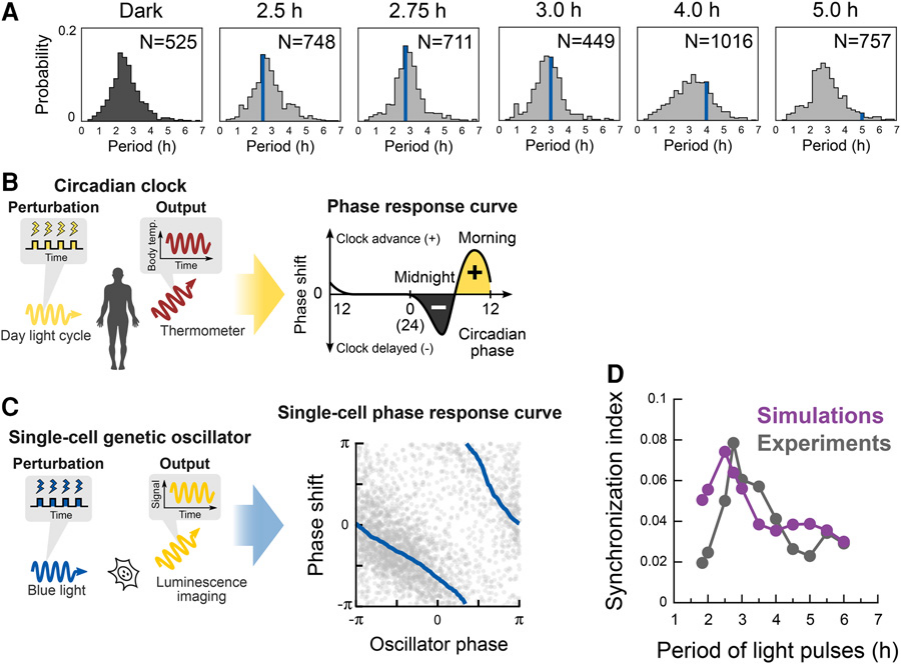
Stochastic phase model reproducing the dynamic responses of individual oscillators. (*A*) Distributions of peak-to-peak intervals under the dark condition or periodic perturbation at periodicities ranging from 2.5 to 5.0 h. A blue line in the graphs represents the period of light treatment. (*B*) Schematic diagram of perturbation experiments of circadian clocks to yield phase response curves. (*C*) Schematic diagram of perturbation experiments of single-cell oscillators and single-cell phase response curves. Gray dots represent phase shift events (*n* = 2725) collected from data of single-cell perturbation experiments. A moving average is shown by the solid blue lines. (*D*) Synchronization index obtained by numerical simulations of a stochastic phase equation (purple), recapitulating experimental observations (gray).

To better understand the dynamic responses of the oscillators, we computed probability distributions of the period of oscillations. Under cyclic illumination with a 2.5- to 3.0-h period, the peak positions of the distributions were close to the periods of external forcing (Fig. 4A, blue vertical lines), suggesting that the majority of oscillators adapted to the periodicity of the external perturbation. Moreover, even in light conditions with a 4.0-h period that does not match the intrinsic period of the oscillators, some of the oscillators adjusted to the periodicity of the external forcing (Fig. 4A). Together, these observations indicate that *Hes1* oscillators not only maintain their intrinsic period but can also adapt to the periodic inputs by modulating their own periods.

The above results raise the question of what causes individual cells to adapt to a periodic external stimulus or persist in their intrinsic cycling dynamics. To investigate the mechanism by which single-cell genetic oscillators solve these contradictory requirements for persistence and adaptation, we tested a phase model that uses a simple mathematical form to describe the temporal dynamics of self-sustained oscillators and their responses to external perturbation (Winfree 2001). Phase models have been used to study dynamic responses of nonlinear oscillators in diverse situations, such as circadian rhythms, neuronal firing, and heartbeats (Pikovsky et al. 2001; Winfree 2001; Granada et al. 2009). In the case of circadian rhythms in humans, daylight illumination in the morning advances the phase of the body clock and gives rise to adaptation and entrainment of circadian rhythms to the daylight cycle (Fig. 4B). Quantitative rules for the modulation of oscillatory states upon external perturbation can be characterized by phase response curves, which represent a key mechanism for robust and tunable responses of oscillators. This mechanism might also be applicable to the single-cell dynamics in the *Hes1* oscillators (Fig. 4C).

We derived a single-cell phase response curve from the luminescence traces of individual cells under conditions of light pulses with longer periods than the *Hes1*-intrinsic period (2.57 h) (Supplemental Fig. S5C). Data plots comprising individual events of single-cell phase shifts were highly scattered, indicating the stochasticity of individual oscillators (Fig. 4C, gray dots in the right panel). In sharp contrast, the moving average revealed a quantitative relationship; i.e., the single-cell phase response curve (Fig. 4C, blue lines in the right panel). For example, light illumination at an oscillator phase of π/2 delays the oscillation by almost π, whereas illumination at the phase of π does not alter the phase, suggesting that the dynamic phase shifts of single-cell *Hes1* oscillators are highly dependent on the timing of blue-light illumination. A phase transition curve constructed from the single-cell phase response curves shows the relationships between old and new phases after external perturbation and demonstrates the entrainment process based on an iterated map of the phase dynamics (Supplemental Fig. S5D; Winfree 2001; Granada et al. 2009). These observations provide quantitative insights into the decoding process of single-cell oscillators after external perturbation, showing that *Hes1* oscillators can modulate their own phases and periods, as in the phase response curves, in the presence of a dynamic signaling input.

To further understand how genetic oscillators are entrained to periodic stimuli, we performed numerical simulations of oscillatory dynamics using a stochastic phase model (Supplemental Material, Numerical Simulations; Pikovsky et al. 2001). This stochastic phase model is composed of a small number of parameters related to a natural oscillation period, phase response curves, and dynamical noise. To demonstrate entrainment of Hes1 dynamics, we used experimental results for mean variables of oscillation period and phase response curves. Therefore, our model involves three control parameters: noise intensity, deviation of natural period, and deviation of phase response curve. We computed synchronization indexes in various periods of external stimulation, reproducing the resonant-like behaviors of oscillators observed in the experiments within a proper range of the control parameters (Fig. 4D; Supplemental Fig. S6). Together, these results suggest that the stochastic phase model is sufficient to recapitulate the dynamic behaviors of *Hes1* oscillators in response to temporal perturbations.

### Entrainment of the endogenous *Hes1* oscillation by Dll1 inputs

**Figure 5.**
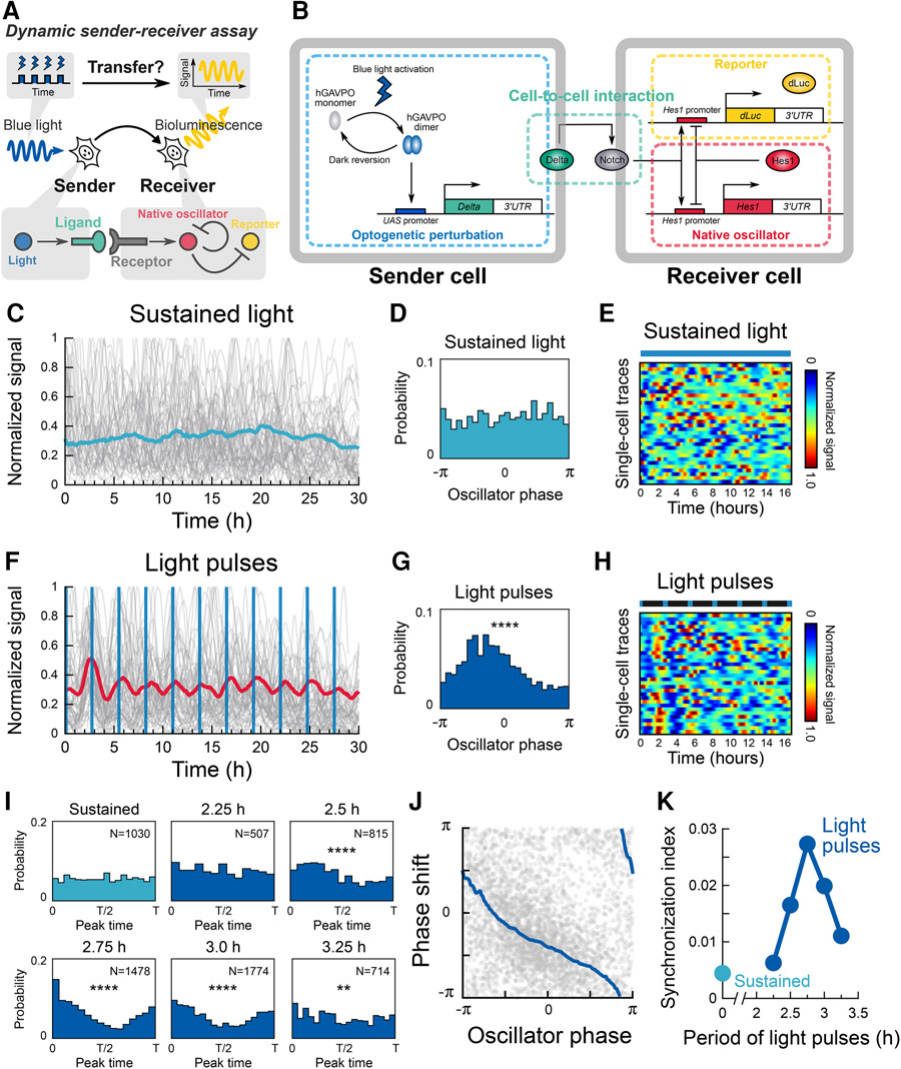
Reconstitution of dynamic cell-to-cell information transfer in genetic oscillators. (*A*) Schematic of a dynamic sender–receiver assay based on a single-cell optogenetic perturbation. (*B*) Schematic of the genetic network comprising cell-to-cell interaction of the Notch signaling pathway. A sender cell carries an optogenetic perturbation module using hGAVPO, while a receiver cell carries a destabilized bioluminescent reporter (dLuc) under the control of the *Hes1* promoter and native oscillator module of the Hes1 negative feedback loop. Sender cells express Dll1 (Delta) by blue-light stimulation. (*C*) Sustained light illumination failed to entrain *Hes1* oscillation in receiver cells. (*D*) Peak time distributions with sustained light illumination. *P* = 0.08, Rayleigh test. (*E*) Heat maps represent single-cell traces with sustained light. (*F*) Entrainment of *Hes1* oscillation in receiver cells by periodic perturbation of sender cells. Blue vertical lines represent the timings of illumination of a 2-min duration. (*G*) Peak time distributions with light pulses. (****) *P* < 0.0001, Rayleigh test. (*H*) Heat maps represent single-cell traces with light pulses. Blue markers in a bar indicate the timing of light illumination. (*I*) Peak time distributions. (****) *P* < 0.0001; (**) *P* < 0.01, Rayleigh test. (*J*) Single-cell responses (gray dots; *n* = 3193 events) and the moving average (blue lines) are shown. (*K*) Synchronization index computed by the entropy-based method.

Having established the dynamic decoding of *Hes1* oscillators, we next asked whether these oscillators can transmit dynamic information to other oscillators. Because oscillations of Hes1 and its related genes drive the oscillatory expression of the Notch ligand Dll1, the Delta–Notch pathway has been functionally implicated in cell–cell transfer of the oscillatory information (Jiang et al. 2000; Okubo et al. 2012; Bone et al. 2014; Soza-Ried et al. 2014; Shimojo et al. 2016). However, there is no experimental evidence for the functional capabilities of Notch signaling molecules to transmit oscillatory information to adjacent cells. We reasoned that oscillatory induction of the Notch ligand Dll1 might be sufficient to convey dynamic information. To test this hypothesis, we sought to reconstitute dynamic cell-to-cell information transfer through a light-inducible Notch signaling pathway (Fig. 5A). We produced photosensitive sender cells that carry an optogenetic perturbation module for Dll1 induction (Fig. 5B, optogenetic perturbation; Supplemental Fig. S7A) and photo-insensitive receiver cells that have the visualization module for *Hes1* promoter activity (Fig. 5B, reporter; Supplemental Fig. S7A). C2C12 cells do not express a meaningful level of Dll1 (wild type in Supplemental Fig. S7C), but the photosensitive sender cells were able to express Dll1 mRNA and protein in a pulsatile manner under light stimulation (Supplemental Fig. S7B–E). The photosensitive sender and photo-insensitive receiver cells were cocultured (Supplemental Fig. S8A), and bioluminescence signals were monitored after blue-light illumination (Supplemental Movies S4, S5). We found that different ratios (2:1, 1:1, 1:2, and 1:5) of receiver versus sender cell mixtures induced synchronized oscillation in receiver cells (Supplemental Fig. S8B). We decided to use the 1:5 ratio for further analyses because it exhibited the largest trough to peak ratio (Supplemental Fig. S8B). When the cells were exposed to sustained light illumination (5-sec duration with 5-min interval between successive illuminations), *Hes1* gene expression dynamics in the receiver cells showed an asynchronous oscillatory pattern (Fig. 5C–E; Supplemental Movie S4). In contrast, periodic blue-light illumination (2-min duration with 2.5- to 3.25-h intervals) induced synchronized oscillation of receiver cells with the same periodicity as the external perturbation (Fig. 5F–I; Supplemental Movie S5). These results showed that the temporal information of the stimulus period was transferred from the photosensitive sender cells to the photo-insensitive receiver cells. Interestingly, Dll1 inputs at 5-h periodicity induced bimodal pulses of Hes1 expression, suggesting resonant-like responses by the oscillators (Supplemental Fig. S8C). However, periodic stimulation with 2.25-h intervals failed to induce apparent directional distribution of peak timings, suggesting that there is a window for transferrable periods (Fig. 5I).

A single-cell time-series analysis yielded a phase response curve (Fig. 5J) and a phase transition curve (Supplemental Fig. S8D); these quantitative data demonstrated that the single-cell genetic oscillators were responsible for initiating phase modulation depending on the timing of external perturbation in surrounding cells. This indicates that the cell-to-cell communications follow the synchronization scenario described by the stochastic phase model. This scenario was further supported by the resonant-like behavior of the oscillators (Fig. 5K; Supplemental Fig. S8E). Taken together, these findings provide direct evidence that single-cell genetic oscillators can transmit and decode dynamic information in multicellular interactions.

### Measurement of coupling delays in Delta–Notch signaling transmission

**Figure 6.**
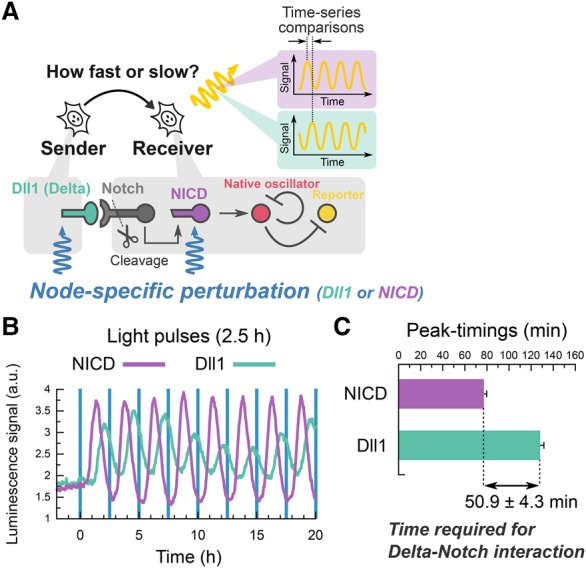
Node-specific optogenetic perturbation reveals the time required for ligand–receptor interactions. (*A*) Schematic of the node-specific perturbation in the Delta–Notch pathway. Membrane-bound Dll1 triggers the cleavage of Notch receptors and gives rise to the production of the NICD, which is an active form of Notch receptor. Node-specific perturbation directly controls either Dll1 or NICD production. (*B*) Population signaling traces of *Hes1* promoter activity in the presence of Dll1-specific (green) or NICD-specific (purple) perturbation with a 2.5-h period. The data were acquired by a photomultiplier tube. Blue vertical lines represent the timings of illumination of a 2-min duration. (*C*) Comparison of peak timings between Dll1-specific (128.1 min ± 3.4 min) and NICD-specific (77.2 min ± 2.6 min) perturbations identified the time required for Delta–Notch signaling transmission (50.9 min ± 4.3 min). *n* = 12 pairs of comparative peaks.

It has been shown that, depending on the coupling delays between cells, gene expression dynamics may change: Oscillations could be in-phase or anti-phase between cells or may be quenched (Ramana Reddy et al. 1998; Shimojo et al. 2016), suggesting that measurement of coupling delays is very important to analyze the expression dynamics. For example, it was shown that both decrease and increase in coupling delays of Delta–Notch signaling dampen in-phase oscillations in the PSM and anti-phase oscillations in neural stem cells, causing defects in somitogenesis and neurogenesis, respectively (Shimojo et al. 2016). In Notch signaling, Dll1 interacts with Notch in neighboring cells in which the Notch intracellular domain (NICD) is then formed and up-regulates *Hes1* expression. Thus, the coupling delays of Notch signaling are involved in the formation of NICDs via Dll1–Notch interaction. Our method offers a powerful tool to measure the time required for these processes (Fig. 6A), which can be measured by monitoring Hes1 responses to extracellular Dll1 inputs and intracellular NICD inputs.

Oscillatory inputs with a 2.5-h period of intracellular NICDs (Supplemental Fig. S9A,B) also induced *Hes1* oscillation in a synchronized manner (Fig. 6B). However, although expression of both Dll1 and NICD occurred at 0.5 h and peaked at 1.0 h after light stimulation (Supplemental Fig. S9C,D), the *Hes1* peaks appeared at different timings between intracellular NICDs and extracellular Dll1 inputs (Fig. 6B). The *Hes1* peaks appeared 128.1 min ± 3.4 min after optogenetic induction of extracellular Dll1 and 77.2 min ± 2.6 min after that of intracellular NICDs (Fig. 6B,C). These data indicate that the time required from Dll1 induction to NICD formation was calculated to be 50.9 min ± 4.3 min in C2C12 cells (Fig. 6C). These results demonstrate this optogenetic perturbation approach as a powerful tool to measure the time required for signaling transfer between cells.

## Discussion

### Integrated approach to control and monitor gene expression patterns with high temporal accuracy

**Figure 7.**
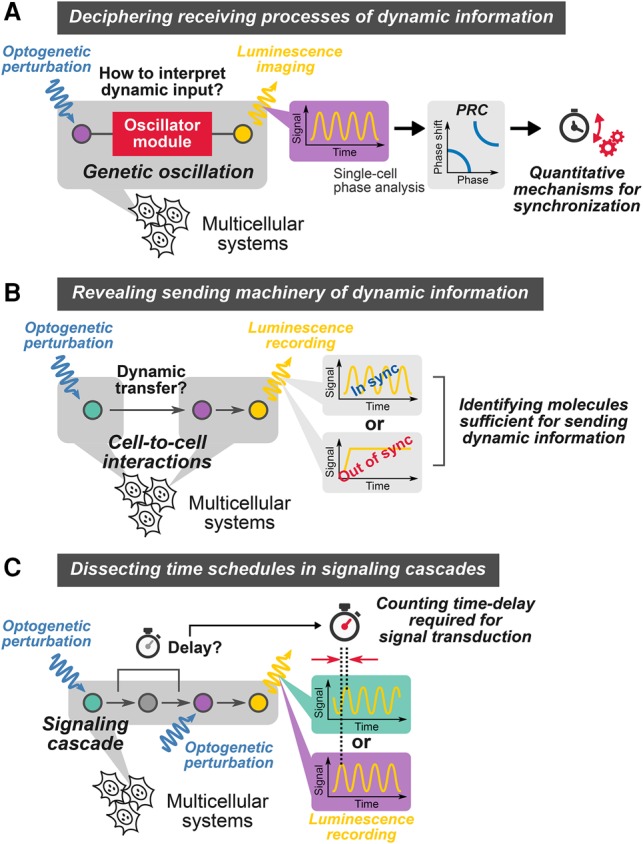
A versatile platform to control and monitor dynamic cell-to-cell transfer of oscillatory information. (*A*) Optogenetic interrogation of receiving processes. (*B*) Optogenetic interrogation of sending processes. (*C*) Optogenetic interrogation of delays required for signal transduction.

In the present study, we developed an integrated and versatile approach that combines optogenetic perturbations and single-cell live imaging with bioluminescence reporters to examine signal-sending and signal-receiving processes of oscillatory gene expression (Fig. 7). One of the unique features of our technology is the high temporal accuracy, which is able to generate pulse trains of gene expression with as short as 1.83-h periodicity. The key to reach this time scale was short half-lives of mRNA products by using unstable 3′ UTRs (Fig. 1B), which were much shorter than those of optogenetic tools used in the previous studies (Wang et al. 2012; Motta-Mena et al. 2014). Because our strategy to induce pulsatile expression is very simple, it is applicable to other gene expression systems.

Another feature is the combination of blue-light-inducible modules with luminescence reporters. We did not observe any decline in signals of luminescence reporters even after repeated illumination of blue light, indicating that this monitoring module is insensitive to photobleaching and is therefore compatible with the optogenetic perturbation module. In contrast, widely used fluorescent reporters, including fast maturating GFP/YFP derivatives and Förster resonance energy transfer (FRET) probes comprising CFP–YFP pairs, are vulnerable to blue-light illumination, which may limit the applicability to blue-light-driven optogenetics. Thus, our method has the advantage compared with ones using fluorescent reporters because there is no undesirable cross-talk between monitoring and perturbation procedures.

### A versatile platform to characterize cell-to-cell transfer of dynamic information

Cells communicate with each other to coordinate their gene expression patterns at the population level, and the communication mechanism has been intensively analyzed. However, conventional methods of sender–receiver assays provide the information about the static ligand activity (amplitude) but not about the frequencies or phases, although it is known that many gene activities are oscillatory and that the frequencies and phases could convey important information for cellular events (Levine et al. 2013; Purvis and Lahav 2013; Isomura and Kageyama 2014). Artificial imposition of signaling factors with a precise temporal schedule, including microfluidics technology, has enabled the characterization of responding factors as information carriers in dynamic gene regulatory programs (Ashall et al. 2009; Gregor et al. 2010; Shalek et al. 2014; Kellogg and Tay 2015). Those methods can manipulate the environment-to-cell stimulus but not the cell-to-cell signaling transmission, and therefore it has proved difficult to examine direct communications between ligand-sending and ligand-receiving cells in a dynamic system.

In our sender–receiver assay (Figs. 5A, 7B), we successfully deciphered the processes in which oscillatory expression of the Notch ligand Dll1 conveyed the oscillatory information to neighboring cells, thereby inducing synchronization at the population level. We also identified a window for transferrable periods of Dll1 oscillation; outside of this window, oscillation did not synchronize between cells. This method is applicable to any gene set of ligands in signal-sending cells and downstream effectors in signal-receiving cells, and thus our new approach offers a unique platform to interrogate the dynamic processes of information transfer in many signaling pathways.

### Elucidation of a quantitative mechanism for cell-to-cell synchronization by frequency tuning and phase shifting

Here, we found that periodic inputs of Notch signaling entrain intrinsic *Hes1* oscillations by frequency tuning and phase shifting at the single-cell level and that oscillatory expression of the Notch ligand Dll1 is sufficient to induce synchronization between neighboring cells. It was shown that when dissociated PSM cells with mixtures of various phases of oscillations were aggregated, they resumed robust and synchronized oscillations within 5–6 h, which is equivalent to two or three cycles, suggesting that synchronization occurs very rapidly (Tsiairis and Aulehla 2016). We successfully measured the phase response curve and phase transition curve for the relationship between Notch signaling inputs and phase shift of Hes1 oscillation in receiving cells. According to the phase transition curve (Supplemental Fig. S8D), when Dll1 inputs begin at phase −2π/3 of Hes1 oscillation, the new phase is also −2π/3. Thus, under this condition, the phase of Hes1 oscillation would not be shifted, suggesting that this timing of Dll1 inputs is suitable for synchronized oscillation. If Dll1 inputs occur π slower than the previous one (at phase π/3 of Hes1 oscillation, which is anti-phase to the previous one), Hes1 oscillation would be shifted to a new phase of −π/4, and when the next Dll1 inputs occur at this phase (−π/4), Hes1 oscillation would be shifted to a new phase close to −2π/3, a condition for no further phase shift (Supplemental Fig. S8D). This result suggests that even the anti-phase state between neighboring cells would become in-phase within two or three cycles. In the systems such as the PSM, both senders and receivers are in the same cells, and therefore synchronization could occur even more rapidly in such systems than when senders and receivers are in different cells, which explains well the result of the PSM cell aggregation study (Tsiairis and Aulehla 2016). Thus, our new method described here successfully elucidates the underlying mechanism of Notch signaling-mediated robust and rapid synchronization.

### Machinery for decoding information in gene regulatory circuits

Oscillatory gene expression exhibits characteristic periods in a context-dependent manner, typically ranging from 1- to 4-h periodicity in mammalian cells (Levine et al. 2013; Purvis and Lahav 2013; Isomura and Kageyama 2014). Previous studies demonstrated that artificial oscillatory inputs of various factors at certain periods are able to trigger downstream biological events (Ashall et al. 2009; Aoki et al. 2013; Imayoshi et al. 2013; Mitchell et al. 2015), raising a question of how single cells sense the input frequency to maximize their responses.

We found that the efficiency of entrainment by direct inputs of Hes1 oscillation is highly dependent on the period (or frequency) (Fig. 3C; Supplemental Fig. S5B). It is noteworthy that the best condition for *Hes1* synchronization (2.75-h periodicity) was close to its natural period of oscillation (2.57 h ± 0.96 h), suggesting that Hes1 oscillators can sense the input frequencies (Fig. 7A). These features can be reproduced by numerical simulations of the stochastic phase model that requires the information about period distributions and phase response curves. Because such information is accessible from the optogenetic perturbation and single-cell imaging experiments, our framework may be applicable to the analysis of other oscillatory gene circuits.

The frequency sensing of external inputs was also observed in circadian clocks; the phase model has been tested extensively at the single-cell resolution to the in vivo levels (Winfree 2001; Ukai et al. 2007; Yamaguchi et al. 2013). Because we were able to recapitulate the dynamic responses of Hes1 oscillations by the phase model with stochasticity, circadian rhythms and Hes1 oscillation are likely to share a common mathematical basis for the clock-tuning dynamics despite the highly different complexity of the circuit structures and the characteristic time scales (2–3 h vs. 24 h). Thus, the phase model may be useful to understand a general mechanism to implement the frequency-dependent response in gene regulatory circuits.

Another feature of Hes1 oscillation distinguishable from circadian rhythms is a large diversity of frequency distributions in the free-running oscillators (Supplemental Fig. S6A), suggesting the stochastic nature of Hes1 oscillation, which is similar to isolated PSM cells (Webb et al. 2016). These observations directed us to take into account the noise effects in our model to reproduce the dynamic responses of single-cell oscillators. Previous reports identified critical roles of noise to simulate dynamic responses of genetic oscillators (Mondragón-Palomino et al. 2011; Kellogg and Tay 2015). We also found that incorporation of the noise effects is essential for satisfactory reproduction of the experimental data (Supplemental Material, Numerical Simulations). These results support the notion that the stochastic nature of gene expression is highly involved in not only free-running oscillations but also responding dynamics.

### Identification of time schedules in signaling cascades

It has been suggested that the coupling delays between cells regulate gene expression dynamics: Oscillations could be in-phase or anti-phase between cells or may be quenched, depending on the coupling delays (Ramana Reddy et al. 1998; Shimojo et al. 2016). Thus, it is important to measure the time schedules in signaling cascades to understand the gene expression dynamics. A recent optogenetic approach revealed the transmission delay in the ERK–Ras signaling pathway by combining single-node-specific optogenetic perturbation (opto-SOS) with fluorescence reporters that monitor localizations of two molecular components: SOS and ERK (Toettcher et al. 2013). However, such desirable pairs of node-specific reporters are not always available, especially in the Delta–Notch signaling pathway.

Here, we developed an alternative method with multiple node-specific optogenetic perturbations, rather than multiple node monitoring, to estimate the transmission delay in Delta–Notch transmission (Figs. 6A, 7C). This novel approach allowed cancellation of the time required for optogenetic activation by subtracting the delays for downstream (NICD in this study) perturbation from the ones for upstream (Dll1 in this study) perturbation, leading to the precise estimation of the delay in Delta–Notch signaling transmission (50.9 min ± 4.3 min) (Fig. 6C). These results demonstrated the utility of our strategy to measure time schedules in signal transmission that comprises invisible steps to conventional reporter systems. Further expansions of node-specific optogenetic perturbation together with the development of other reporters, such as the luciferase complementation-based reporter (Ilagan et al. 2011), will contribute to dissection of complex regulatory mechanisms in the Notch pathway (Bray 2016).

Other branches of optogenetic technology enabled direct control of receptor activity by light, which used fusion proteins of light-sensitive modules and proteins of interest; however, engineering such de novo photosensitive proteins requires trial and error for sufficient specificity and efficiency (Chang et al. 2014; Grusch et al. 2014). The optogenetic system used in this study just induces the expression of genes of interest and is therefore free from such obstacles. Thus, our approach is applicable to various types of signaling pathways in which molecular components, such as ligands, receptors, and effectors, are genetically encoded. In summary, our method will open a new opportunity to decipher the underlying mechanism of how dynamic gene expression is coordinated at the tissue level through cell-to-cell communication.

## Materials and methods

Detailed experimental procedures are provided in the Supplemental Material.

### Plasmid construction (Supplemental Table S1)

All plasmids were based on the Tol2 transposon vector system (Kawakami 2007). Schematic structures are indicated in Supplemental Figures S1A, S2B, S7A, and S9B.

### Generation of stable cell lines (Supplemental Table S2)

Stably transfected cells, which were mCherry- or iRFP-positive, were collected by FACS, and stable lines were picked from isolated colonies.

### Cell culture and time-lapse microscopy

Cells were plated on glass bottom dishes, and luminescence signals were captured by cooled CCD camera (iKon-M 934, Andor). Each blue-light pulse was delivered for 2 min.

### Image processing and time-series analysis

Images were processed by ImageJ image analysis software.

### Synchronization index and Kuramoto order parameters

We assessed the level of synchronization using a synchronization index and Kuramoto order parameters for the peak time distribution.

### Constructing phase response curves and phase transition curves

Phase response curves were constructed using single-cell phase traces computed by Hilbert transformation. Phase transition curves were obtained by mapping the time series of phase θ (*t*) to an old phase and θ (*t* + *T*_0_) to a new phase.

### Live cell monitoring of luminescence signals by a photomultiplier tube

Luminescence was recorded by a highly sensitive photomultiplier tube with a LED blue-light source (Churitsu Electric Corp., CL24B-LIC/B). Each blue-light pulse was delivered for 30 sec. The duration of the light pulse was set shorter than that used for time-lapse microscopy because responses at the population level were easier to detect.

### Numerical simulations

We used a stochastic phase model, which involves three control parameters: the noise intensity, the deviation of the natural period, and the deviation of the phase response curve.

## Supplementary Material

Supplemental Material
