# Supplementary material for: Optogenetic perturbation and bioluminescence imaging to analyze cell-to-cell transfer of oscillatory information
Source: Genes Dev. 2017 Mar 1;31(5):524–35. doi: 10.1101/gad.294546.116 (PMC5393066; doi:10.1101/gad.294546.116)
Supplement: Supplemental Material [file supp_31_5_524__index.html]

Optogenetic perturbation and bioluminescence imaging to analyze cell-to-cell transfer of oscillatory information — Supplemental Material 

# Optogenetic perturbation and bioluminescence imaging to analyze cell-to-cell transfer of oscillatory information

## Supplemental Material

- Supplemental\_data.pdf
- Supplemental\_Movie\_1.mp4
- Supplemental\_Movie\_4.mp4
- Supplemental\_Movie\_2.mp4
- Supplemental\_Movie\_5.mp4
- Supplemental\_Movie\_3.mp4
