# Supplementary material for: Optogenetic perturbation and bioluminescence imaging to analyze cell-to-cell transfer of oscillatory information
Source: Genes Dev. 2017 Mar 1;31(5):524–35. doi: 10.1101/gad.294546.116 (PMC5393066; doi:10.1101/gad.294546.116)
Supplement: Supplemental Material [file supp_gad.294546.116_Supplemental_data.pdf]

Materials and Methods

Plasmid construction

We used standard molecular biology techniques to assemble the constructs used in this study. All plasmids were based on the Tol2 transposon vector system (a gift from the Kawakami Lab (Kawakami, 2007; Yagita et al., 2010)) and details of the plasmid constructions are shown in Figs. S1A, S2B, S7A, S9B, and Table S1.

To construct the Hes1 promoter reporter, the *Hes1 cis*-regulatory motif (CRM5) was fused to the *Hes1* promoter region (-2567 to +223), Ub-NLS-Luc2 reporter and *Hes1* 3′UTR (Figs. S2B, S7A and S9B) (Masamizu et al., 2006; Kobayashi et al., 2009; Jeziorska et al., 2012). We also constructed an expression cassette in which H2B-mCherry was driven by a constitutively active promoter pPGK; the cassette was used for tracking nuclear positions by image processing and for FACS sorting (Carr and Whitmore, 2005). These two modules were used in pAI177 and pAI201 (Figs. S2B, S7A and S9B).

To generate blue-light sensitive modules, we employed the previously described humanized version of GAPVO (termed *hGAVPO*), obtained from GeneArt gene synthesis service (Life Technologies) (Imayoshi et al., 2013). *hGAVPO* coding sequences were fused to a constitutively active pPGK promoter. Each gene of interest (*dLuc*, *Hes1*, *Dll1* and *NICD*) was fused upstream of the *Hes1* 3′UTR and downstream of a UAS promoter derived from the LightOn system (a gift from the Yang Lab). Plasmid pAI189 encodes a red-shifted *Renilla reniformis* luciferase variant, *RLuc8.6-535* (a gift from the Gambhir Lab) (Loening et al., 2007), as a marker gene for clonal selection (Figs. S2B, S7A and S9B). Another plasmid, pAI235, contains a coding sequence of a tandem version of an infrared protein, iRFP713 (a gift from the Michiyuki Matsuda Lab) (Filonov et al., 2011), and was used in FACS sorting.

Generation of stable cell lines

To establish stable cell lines (Table S2), 0.5 μg pT2A and 0.5 μg pCAGGS-mT2TP plasmids were transfected using Lipofectamine LTX reagent (Life Technologies) into C2C12 cells cultured in a 12-well plate with 5×10^4 cell-density. Transfected cells were expanded and drug-selected with either 300 μg/ml G418 or 100 μg/ml Hygromycin. Stably transfected cells, which were mCherry- or iRFP-positive, were collected by FACS and clonal lines were picked from single isolated colonies. To identify photo-sensitive oscillator cell clones or photo-activatable oscillator cells, we selected clones that displayed luminescence activity due to *R. reniformis* luciferase (Figs. S2B and S9B).

Cell culture and time-lapse microscopy

C2C12 myoblast cells were maintained in DMEM medium supplemented with 10% FBS and penicillin/streptomycin. The cells were cultured at 37°C and 5% CO_2 in a humidified incubator. For time-lapse microscopy, cells were plated on a 27-mm diameter glass bottom dish (IWAKI) in DMEM medium containing 10% FBS, 1 day before observation. For the optogenetic perturbation assay (Fig. 2A), 1×10^5 photo-sensitive cells and 1.5×10^5 feeder cells were co-plated. For the dynamic sender-receiver assays (Fig. 5), 5×10^4 photo-insensitive receiver cells and 2.5×10^5 photo-sensitive sender cells were co-plated. The medium was exchanged with phenol red-free DMEM medium containing 5% FBS and 1mM luciferin before observation. We captured single-cell time-lapse movies by using an inverted microscope (IX81-ZDC, Olympus) equipped with an environmental chamber at 37 °C and 5% CO_2, a ×40 objective (UPLFLN 40XO, N.A. 1.30, Olympus), a cooled CCD

camera (iKon-M 934, Andor) and automated acquisition software (Metamorph, Molecular Devices). Luminescence and fluorescence images were acquired using 4×4 binning with 50 kHz read-out mode and 2×2 binning with 1 MHz read-out mode, respectively. Time-lapse images were collected at 5-min intervals with 270-sec exposures of the luminescence channel and 400-msec exposures of the fluorescence channels. For fluorescence imaging, 565-nm and 635-nm LED light sources were used for excitation of mCherry and iRFP713 fluorescent proteins, respectively. Blue-light pulses were delivered by turning on the excitation light for GFP fluorescence (470-nm blue LED light source, an excitation filter 500 nm / 20 nm, and a dichroic beam splitter 515 nm) with duration of 2 min and power intensity of 1.55×10^2 W/m^2 measured by a light meter (S170C and PM100D; Thorlabs).

Image processing and time-series analysis

We employed image processing and time-series analysis techniques to quantify oscillatory dynamics at the single-cell level. Images were processed by ImageJ image-analysis software with custom plug-ins written in Java. The extracted time-series of luminescence signals were further analyzed by custom-made scripts written in Python. These custom written programs are available upon request.

To analyze luminescence time-lapse movies, we filtered out hot pixels derived from cosmic rays by comparing the intensities of pixels in temporally adjacent images, replacing the pixel values to the temporal average of the previous and next frames (implemented as a custom written "SpikeNoise Filter" plug-in for ImageJ). After the removal of hot pixels, we applied a spatially smoothing filter (the "Sigma Filter" plug-in for ImageJ) to the processed images, then subtracted background pixel values of out-field views from every frame.

We also analyzed images of H2B-mCherry fluorescence to detect regions of interest in individual cell nuclei. To this end, we applied local thresholding binarization methods, using a custom written "Local Thresholding Auto-segmentation" ImageJ plug-in, to the mCherry-fluorescence images, yielding binarized time-lapse movies of cell nuclei.

To extract single-cell traces of luminescence signals, we implemented an automatic tracking program (termed "Dynamic Particle Analyzer") for ImageJ plug-in written in Java. This allowed us to identify nuclear regions from the binary images and extract luminescence signals by mapping nuclear regions onto luminescence images. The plug-in automatically analyzed positional movements of nuclei by connecting two nuclei in adjacent frames to construct trajectories for individual cells. We manually checked all the processed trajectories and removed incorrectly connected trajectories.

Single-cell traces of phase dynamics were obtained by applying a temporal smoothing filter (Savitzky-Golay filter, order 2, window 13), moving-average detrending (with 40 frames temporal window) and Hilbert transformation (Pikovsky et al., 2001), to the single-cell traces of luminescence signals extracted in the previous step (Figs. S4A and S4B). Note that the length of time-series is shortened during the procedures of moving-average, because we cannot calculate averaging values at the first and the last 20 frames. This shortening causes blank frames in phase maps as shown in white colors in Fig. S4E. To quantify periods of oscillators, peaks in the single-cell luminescence time series were identified by referring to the oscillator phases computed by the Hilbert transformation (see also Figs. S4A and S4D), i.e. a peak was marked when the oscillator phase corresponds to zero, and peak-to-peak intervals were counted to construct histograms of period distributions (Fig. 4A).

To evaluate whether a population of oscillators were synchronized, we applied the Rayleigh test to the phase distributions constructed from the single-cell traces of the phase

information (Figs. 2G, 2H, 5D and 5G).

Synchronization index and Kuramoto order parameters

We assessed the level of synchronization using a synchronization index (Tass et al., 1998; Mondragon-Palomino et al., 2011; Lin et al., 2012; Teng et al., 2013) and Kuramoto order parameters (Pikovsky et al., 2001) for the peak-time distributions (Figs. 3B and 5I). The synchronization index ρ, based on the Shannon entropy S of the peak-time distribution, is defined by:

$$\rho = 1 - \frac{S}{S_{\max}}, \tag{1}$$

where $S = -\sum_{k=1}^{N} p_k \ln p_k$, p_k is the relative frequency of finding peak times within the k-th bin, N is the number of bins, and $S_{\max} = \ln N$ is the maximum entropy. ρ assumes 0 for a uniform distribution and 1 for a distribution localized in a bin.

To deal with 1:1 and 1:2 synchronization, we defined two types of Kuramoto order parameters. By 1:1 and 1:2 synchronization, we mean that the peak time distribution had one peak and two peaks, respectively. The former and the latter correspond to the situation where every oscillator typically oscillates once or twice within one period of light stimulation, respectively. The Kuramoto order parameters are defined as:

$$R_1 = \frac{1}{M}\left|\sum_{j=1}^{M} e^{i2\pi t_j/T}\right|, R_2 = \frac{1}{M}\left|\sum_{j=1}^{M} e^{i4\pi t_j/T}\right|, \tag{2}$$

where $i = \sqrt{-1}$ is the imaginary unit, t_j is a peak time ($j = 1, 2, \ldots, M$), M is the total number of peak times, and T is the period of light stimulation. Note that $R_1 = 1$ when all t_j values are identical. On the other hand, $R_2 = 1$ when all t_j values are the same or a half, and the other half of t_j values have identical values separated by $T/2$. Both R_1 and R_2 assume 0 when t_j is uniformly distributed within $(0, T)$. Therefore, R_1 and R_2 quantitatively characterize the level of 1:1 and $1:2$ synchronization, respectively (Fig. S5B).

Constructing phase response curves (PRCs) and phase transition curves (PTCs)

PRCs were constructed using single-cell phase-traces computed by Hilbert transformation as described above. We obtained the phase-shift by calculating $\theta(t + T_0) - \theta(t)$, where $\theta(t)$ is the oscillator phase at the time of one light stimulation (t) and T_0 is the natural period of Hes1 oscillation (Fig. S5C). Because the luminescence signals were sampled every 5 min for the exposure of the CCD camera, the time-series of phase θ were also obtained in a discretized manner with 5-min intervals. Thus, we set the natural period T_0 to 155 min that is a multiple of 5 min and close to the observed natural period, 2.57 h (Figs. 4A and S6A). We also set the period of blue light stimuli (T) to be longer than T_0. Because the phase dynamics of Hes1 oscillation fluctuates and diffuses during one stimulation interval of T, the next stimulation was able to hit the phase $\theta(t + T)$ differing from $\theta(t)$. This allowed us to repetitively sample phase-shift events in a single time-lapse movie. We obtained PTCs (Figs. S5D and S8D) by mapping $\theta(t)$ to an old phase and $\theta(t + T_0)$ to a new phase.

Live cell monitoring of luminescence signals by a photo-multiplier tube (PMT)

Luminescence signals at the population level were recorded by a live cell monitoring system (CL24B-LIC/B, Churitsu Electric Corp.) equipped with a high-sensitive PMT and a LED blue-light source (LEDB-SBOXH, OptoCode). Cells were plated on black 24-well plates and photon-counting measurements were performed every 3 min with 5 sec exposure. Light stimuli were applied every 2.5 h with 30 sec duration and the intensity of 31.2 W/m^2 measured by a light meter (LI-250A; LI-COR Biosciences) unless otherwise described. Recorded traces were smoothed by a Savitzky-Golay filter (order 4, window 13).

Western blotting analysis

To prepare lysate of photo-induced cells, 1.5×10^5 (1A3 lines) or 1.0×10^5 (D1S2 and 1C lines) cells were plated on 35 mm diameter dishes and maintained in the dark conditioned CO_2 incubators equipped with a LED blue-light source (LEDB-SBOXH, OptoCode) for 2 days until starting light stimuli. In a periodic stimulation experiment (Fig. S7D), cells were maintained in the dark for 1.5 days and then periodic stimuli were started.

The following antibodies were used for Western blotting analysis: rabbit anti-Hes1 antibody (gift from Dr. Tetsuo Sudo), anti-HA-Peroxidase antibody (Roche, clone 3F10), goat anti-Dll1 polyclonal antibody (Santa Cruz, sc-8155), rabbit anti-Actin polyclonal antibody (Sigma, A2066) and anti-Actin-Peroxidase antibody (Wako, clone 2F3). To quantify protein expression levels, βActin bands were used as loading controls in Figs. S3A, S7C, S7D and S9C.

Real-time PCR analysis

To collect RNA samples, lysate of photo-induced cells was prepared as described above. Real-time PCR analysis was performed by StepOnePlus real-time PCR system (Applied Biosystems). The following primers were used for real-time PCR: *Gapdh* forward, AATGTGTCCGTCGTGGATCTGA; *Gapdh* reverse, AGTGTAGCCCAAGATGCCCTTC; *HA-Hes1 3'UTR* forward, CCCATACGATGTGCCAGATTACGC; *HA-Hes1 3'UTR* reverse, ACAAATGCAGTGCATGGTCAGTCAC.

Supplementary Information for Numerical Simulations

We constructed a mathematical model to gain deeper insights into the entrainment process of 1:1 and 1:2 synchronization (Fig. 3C). Although in principle many variables could be included in our system, we were mostly concerned with developing a minimal model that reproduced experimental results, particularly the synchronization index (SI), ρ, and the Kuramoto order parameter, R_1, for different stimulus periods (Fig. 4D). The occurrence of a well-defined PRC suggested the possibility that a phase model, which has only one variable corresponding to the time information in oscillation, might satisfactorily describe Hes1 dynamics. Phase models are known to produce a good approximation of the dynamics of perturbed and coupled oscillators (Kuramoto, 1984; Winfree, 2001; Pikovsky et al., 2001) and have been used to describe, analyze and predict the synchronization of chemical and biological systems (Kiss et al., 2007; Yamaguchi et al., 2013). However, compared with those previous studies, our system involves much stronger fluctuation. Therefore, to quantitatively reproduce our experimental results, it was essential to incorporate appropriate noise terms. After trial and error, we finally came up with the phase model given by:

$$\frac{d\phi_i}{dt} = \frac{2\pi}{T_i} + \sum_{n=1}^{N} Z_{n,i}(\phi_i)\delta(t - nT_e) + \eta W(\phi_i)\zeta(t), \tag{3}$$

where $\phi_i(t)$ and T_i are the phase and the natural period of the i-th cell; T_e is the period of external stimulus; δ is the Dirac delta function; and Z and W are phase response functions (described below); ζ is white Gaussian noise with zero mean and unit variance, and η is noise intensity. We assumed that T_i is normally distributed with mean μ and standard deviation σ_{T_0}. To incorporate the variability in phase responses to external stimuli observed in the single-cell PRC plot (Fig. 4C), we assumed that the response function for light stimuli is n-dependent. Thus, we assumed $Z_{n,i}(\phi_i) = \xi_{n,i}\widetilde{Z}(\phi_i)$, where $\widetilde{Z}(\phi)$ is the average PRC function directly estimated from the experimental data (detailed below), and $\xi_{n,i}$ is a random number independently taken from the von Mises distribution

$$f(\theta, \kappa) = \frac{\exp(\kappa \cos\theta)}{2\pi I_0(\kappa)}, \tag{4}$$

where κ is a parameter determining the circular standard deviation, given as

$$\sigma_{\text{circular}}(\kappa) = \sqrt{-2\ln\left(\frac{I_1(\kappa)}{I_0(\kappa)}\right)}, \tag{5}$$

with $I_0(\kappa)$ and $I_1(\kappa)$ being the modified Bessel functions of the first kind with order 0 and 1, respectively. Note that the von Mises distribution is a probability distribution on a circle, which is the circular analogue of the normal distribution. We also assumed that the multiplicative noise process $W(\phi_i)$ is the same as the phase response function for light stimuli, i.e., $W(\phi_i) = \widetilde{Z}(\phi_i)$.

As a fitting function for the phase response function experimentally obtained, denoted by $\widetilde{Z}_{\text{ex}}(\phi)$, we employed the function derived from an angular regression map (Downs and Mardia, 2002), given by:

$$\widetilde{Z}_{\text{sm}}(\phi) = \beta + \frac{2}{\pi}\tan^{-1}\left\{\omega \tan\frac{1}{2}\left(\phi - \alpha\right)\right\}. \tag{6}$$

where parameters α and β are angular location parameters and $\omega(\in [-1,1])$ is a slope parameter. We determined these three parameters by minimizing the mean square error between $\widetilde{Z}_{\rm sm}(\phi)$ and $\widetilde{Z}_{\rm ex}(\phi)$ with a stimulus of a 2.75-h period to obtain $(\alpha,\beta,\omega) = (-0.28,-0.35,-0.36)$, resulting in a good fit (Fig. S6B). For comparison, we also employed a piece-wise linear function $\widetilde{Z}_{\rm li}(\phi)$ that connects the mean values of $\widetilde{Z}_{\rm ex}(\phi)$ at neighboring windows (symbols in Fig. S6B).

In our numerical simulations, we employed 5,000 independent cells. The phase equation was solved using Euler's method with a constant time step $\Delta t = 0.01$ and a fully random initial condition for ϕ_i. There are still undetermined parameters in our model: the mean μ and the standard deviation σ_{T_0} for the natural period T_i, the strength η of dynamical noise, and κ determining the strength of the response noise. We set $\mu = 2.57$, which is the average period of the Hes1 oscillation under dark conditions (Fig. S6A). The other three parameters were determined to provide the best fit the synchronization index ρ and the order parameter R_1 (Figs. S6C and S6D), resulting in $(\sigma_{T_0},\eta,\kappa) = (0.18, 0.38, 1.875)$ and $(0.02, 0.04, 1.125)$ for the employed phase response functions $\widetilde{Z}_{\rm sm}(\phi)$ and $\widetilde{Z}_{\rm li}(\phi)$, respectively. As shown in Figs. S6C and S6D, our model qualitatively reproduced ρ and R_1. In particular, we obtained two peaks in ρ, corresponding to 1 : 1 and 1 : 2 entrainment, as observed experimentally. Although our optimization was carried out using ρ and R_1, we found that the phase distribution for each period of light stimulation was in a good agreement with experimental one (Fig. S6E). The distribution of the peak-to-peak period was also well reproduced for some T values (Fig. S6F), indicating the occurrence of frequency shifts by periodic stimuli.

Supplementary Discussion for Numerical Simulations

We showed that our simple model could reproduce the experimental results. Here, to obtain the phase response curve in our model, we used only the experimental data of a particular period of light stimulation ($T = 2.75$). Nevertheless, we could reproduce the peak-time distributions for different T values. Therefore, our model helps us to predict the system's behavior for different periods of time stimulation from the knowledge of a particular condition.

We also found that noise terms were essential for a satisfactory reproduction of experimental results. Our model contained three different types of noise: a variation in the natural period (quenched noise), multiplicative noise (dynamical noise), and response noise. The response noise was essential to reproduce the SI while keeping the quantitative behavior of R_1.

A more subtle issue is the combination of quenched and dynamical noises. In our preliminary numerical analysis, we considered the absence of dynamical noise, where the period variability under dark conditions was fully accounted for by the distribution of the natural period. In this case, we could only obtain smaller SI and R_1 values without any clear peak, which obviously differed from those in the experiments.

Such fluctuations originate from various causes, including intrinsic, extrinsic, and observation noises. The importance of intrinsic and extrinsic noises in genetic oscillations has been extensively discussed (see (Eldar and Elowitz, 2010; Mondragon-Palomino et al., 2011; Kellogg and Tay, 2015) and references therein). To our knowledge, there is no consensus on how to incorporate these noises in phase models. Theoretical studies on this topic will be of great importance for understanding of intrinsic dynamics and the response of genetic oscillators.

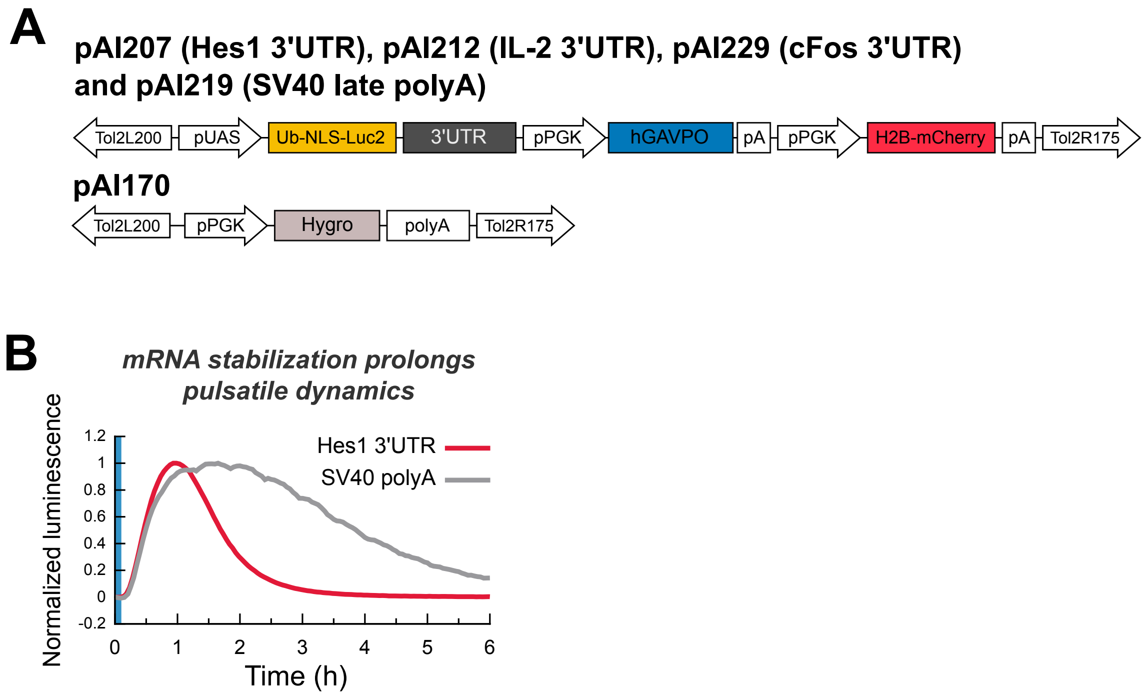

Supplementary Figure 1. Optogenetic perturbation system, Related to Figure 1.
(A) Schematic structures of the plasmid constructs used in Figures 1B-F and S1B. (B) Time-series of bioluminescence signals upon single blue-light stimuli.

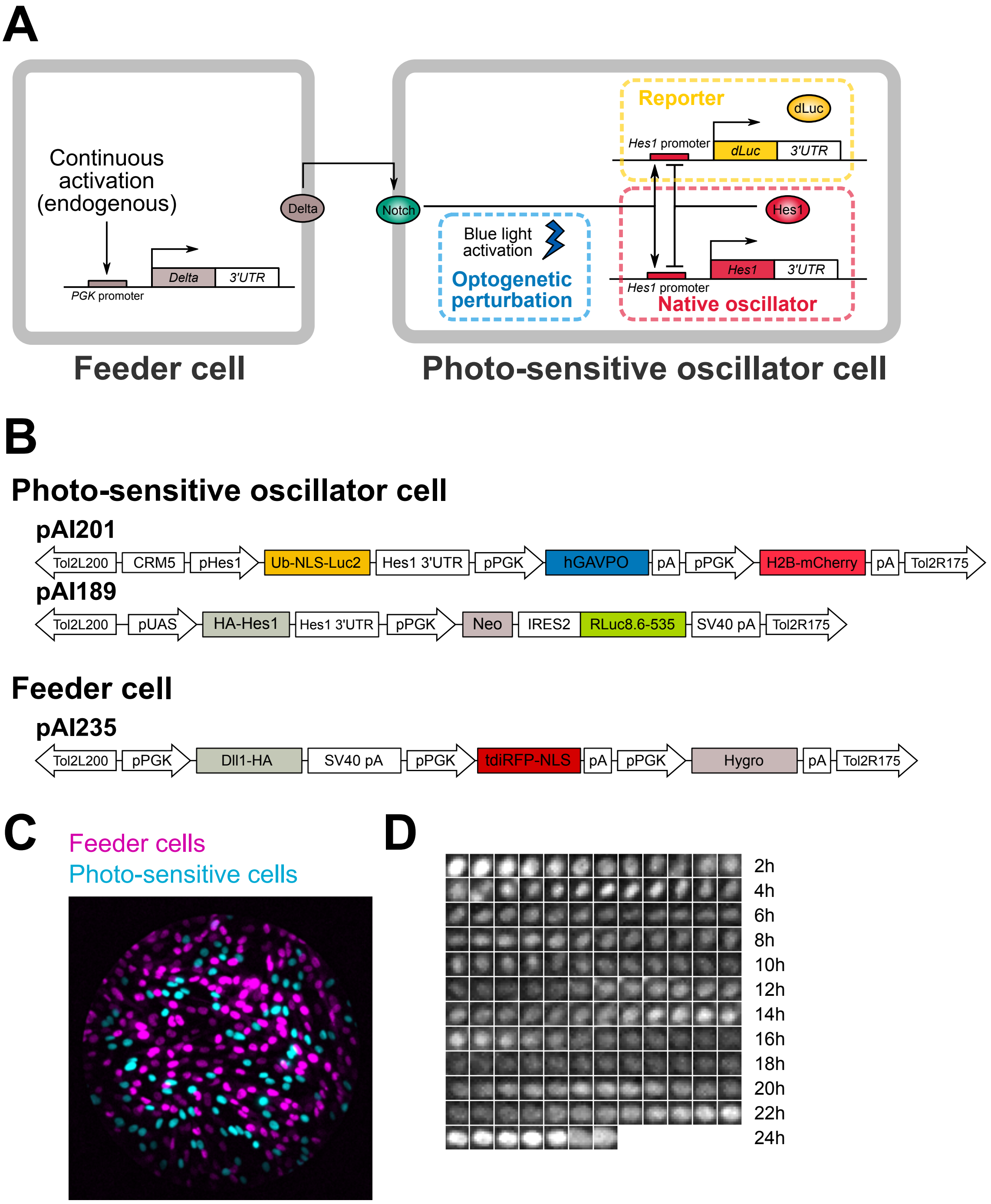

Supplementary Figure 2. Single-cell luminescence imaging of Hes1 promoter activity, Related to Figure 2.
(A) A schematic diagram of co-culture experiments. Photo-sensitive oscillator cells (also shown in Figure 2B) were co-cultured with feeder cells constitutively expressing the Notch ligand Dll1 (Delta). These feeder cells provide sustained activation of the Delta-Notch signaling pathway, thereby driving the negative feedback loop of Hes1 oscillation. A simplified version of the optogenetic perturbation module (detailed in Figure 2B) is shown in this diagram. (B) Schematic structures of the plasmid constructs used for the photo-sensitive oscillator cells and feeder cells. (C) A mixed culture of photo-sensitive oscillator cells labeled with mCherry (cyan) and feeder cells labeled with iRFP713 (magenta). (D) Single-cell snapshots showing oscillatory expression of the luciferase reporter under the control of Hes1 promoter in a dark condition.

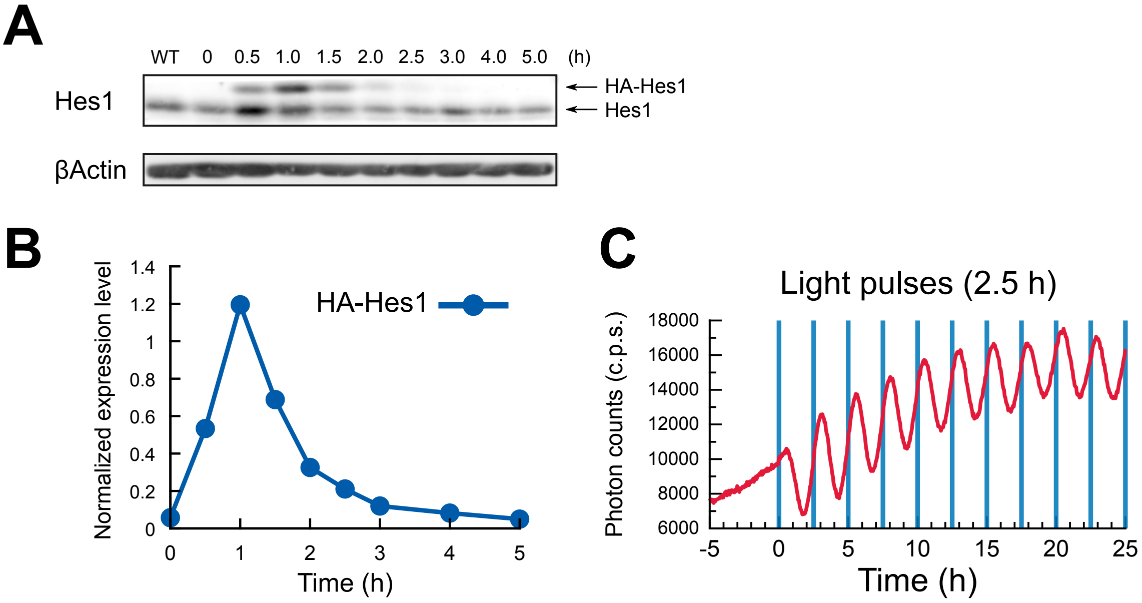

Supplementary Figure 3. Light-induced protein expression of Hes1 in photo-sensitive oscillator cells. Related to Figure 2.

(A) Western blotting analysis of Hes1 expression dynamics induced by single-pulse illumination. A light stimulation was applied with 2 min duration and the intensity of 24.7 W/m^2 measured by a light meter (S170C and PM100D; Thorlabs). (B) Expression levels were normarized by the value of endogenous (WT) Hes1 level in (A). (C) Population signaling-traces of Hes1 promoter activity in the presence of Hes1 perturbation with a 2.5-h period, which were acquired by a photo-multiplier tube. Before starting light illumination, oscillatory patterns were absent at the population level, because of non-synchronous oscillation. Blue vertical lines represent timings of illumination with 30 sec duration. c.p.s., counts per second.

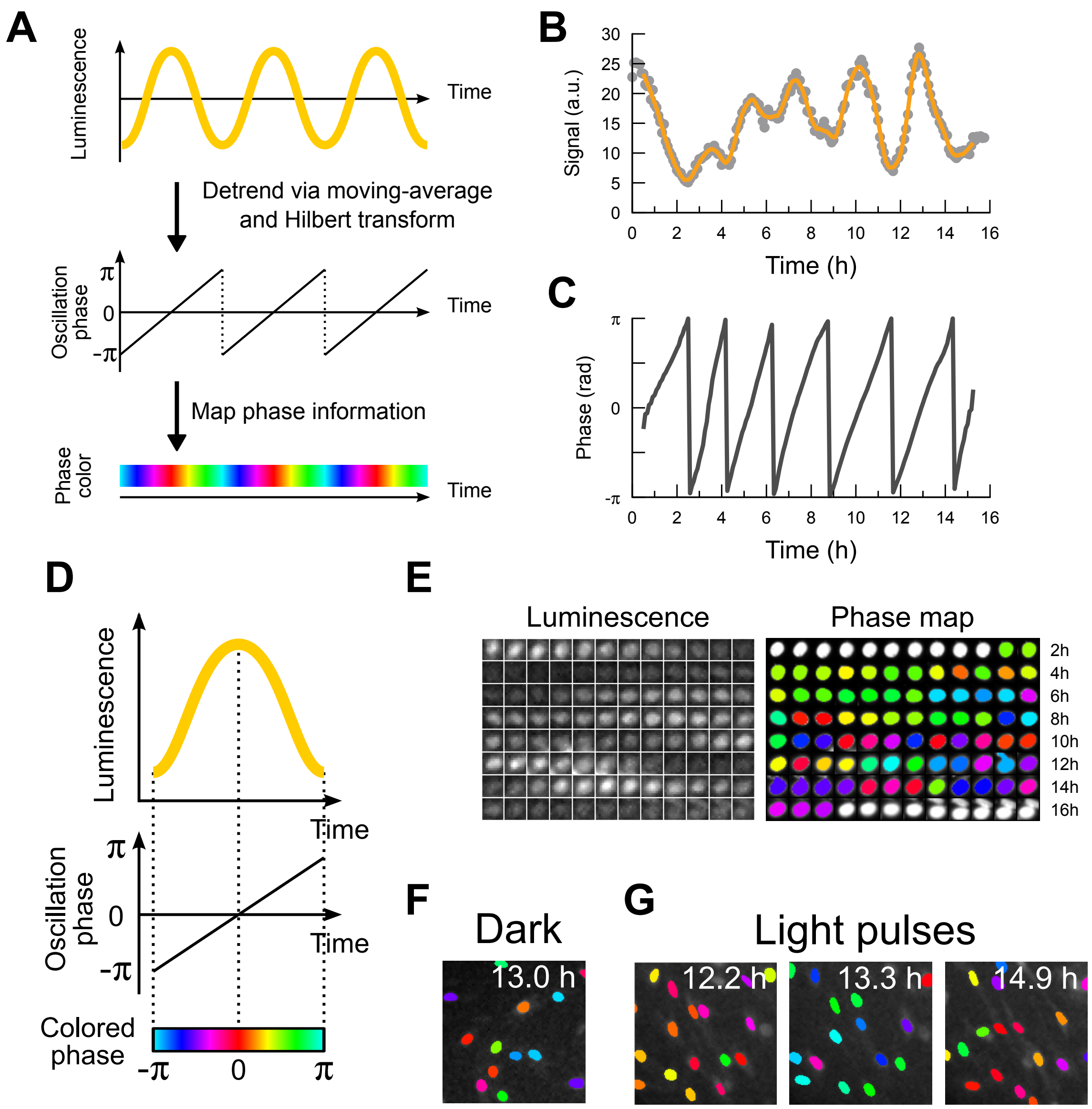

Supplementary Figure 4. Time-series analysis of single-cell luminescence traces, Related to Figure 2.
(A) Schematic outline of the process in the time-series analysis to obtain phase information for individual oscillators. A time-series extracted from luminescence movies is first smoothed by the Savitzky-Golay filter (yellow line), then detrended and Hilbert-transformed to obtain the phase information (black lines). The phase information is then converted to a colored-map: peaks are colored red, whereas troughs are colored blue. (B,C) Representative time-series of luminescence signals (B) and phases (C). (B) Raw signal traces (gray dots) were processed by Savitzky-Golay filter to yield smoothed time-series (yellow lines). (C) Phase calculated by Hilbert transformation of the time-series shown in (B). (D) Color-code used to generate phase maps. (E) Comparative snapshots of luminescence images and phase-mapping obtained from the time-series shown in (B) and (C). White colors in phase maps represent phase-undefined frames due to moving averaging procedures (see also Materials and Methods). (F, G) Representative snapshots of a phase map in the absence (F) or the presence (G) of light pulses. Light pulses were applied with a 2.75-h period. Time indexes correspond to the time-frames in Figure 2C,D.

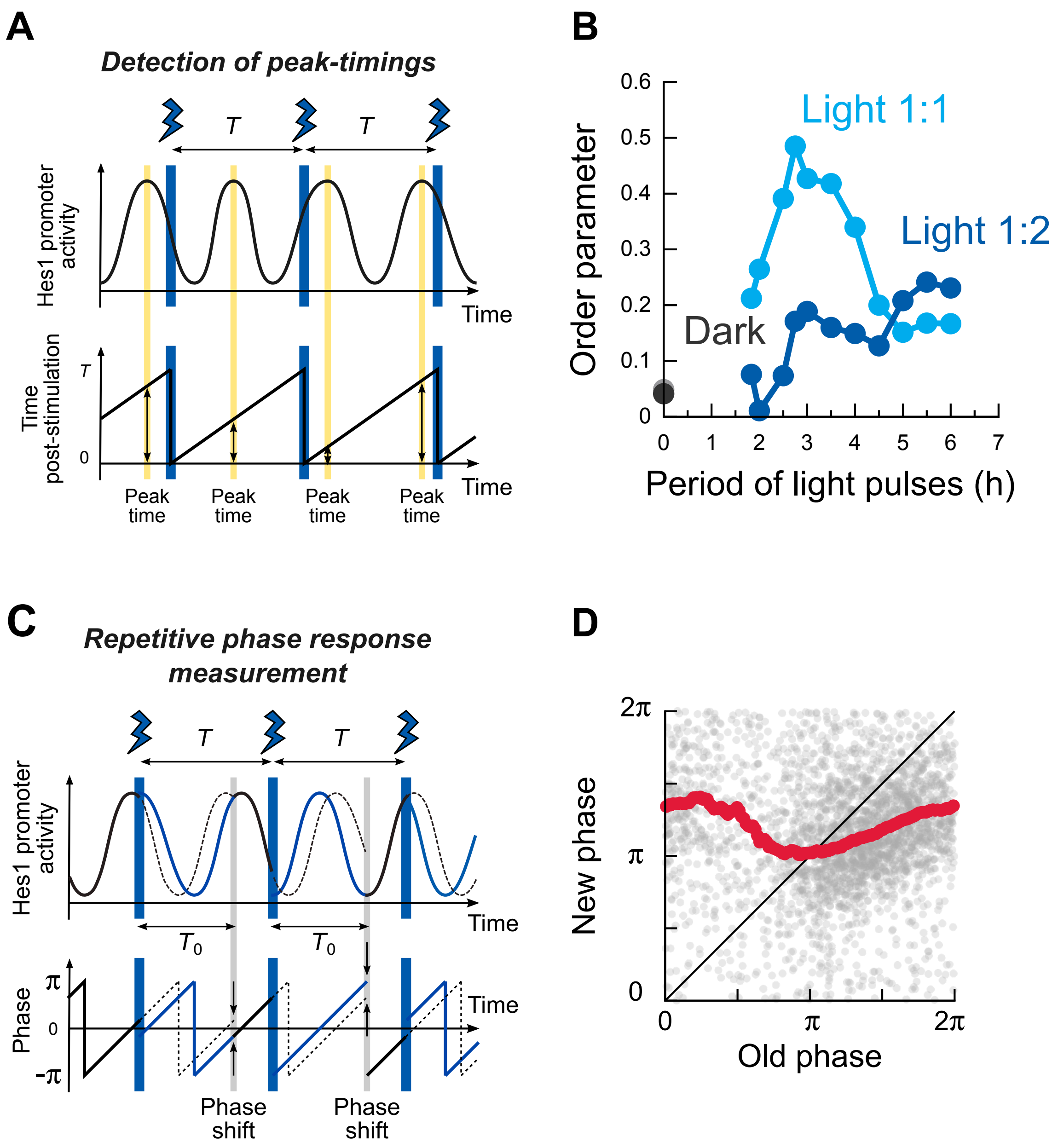

Supplementary Figure 5. Quantification of synchronization efficiency and phase response curves, Related to Figures 3 and 4.
(A) Definition of the peak time (used in Figures 3B and S8D). Note that peaks in the luminescence time series can be identified by referring to the oscillator phases computed by the Hilbert transformation. Yellow vertical lines indicate timings of peaks in single-cell traces. Blue vertical lines represent timings of illumination. (B) Another measure of synchronization efficiency, the Kuramoto order parameter, also represents resonant-like behaviors. (C) Schematic diagram of phase response quantification. Solid lines represent single-cell traces upon optogenetic perturbation. Dashed lines represent false traces. T, a period of blue light stimulus; T_0, the natural period of Hes1 oscillation. (D) A phase transition curve (PTC) computed from the phase response curves in Fig.4C, which represents how the current phases transit to new phases upon external stimulation (Granada et al. (2009)).

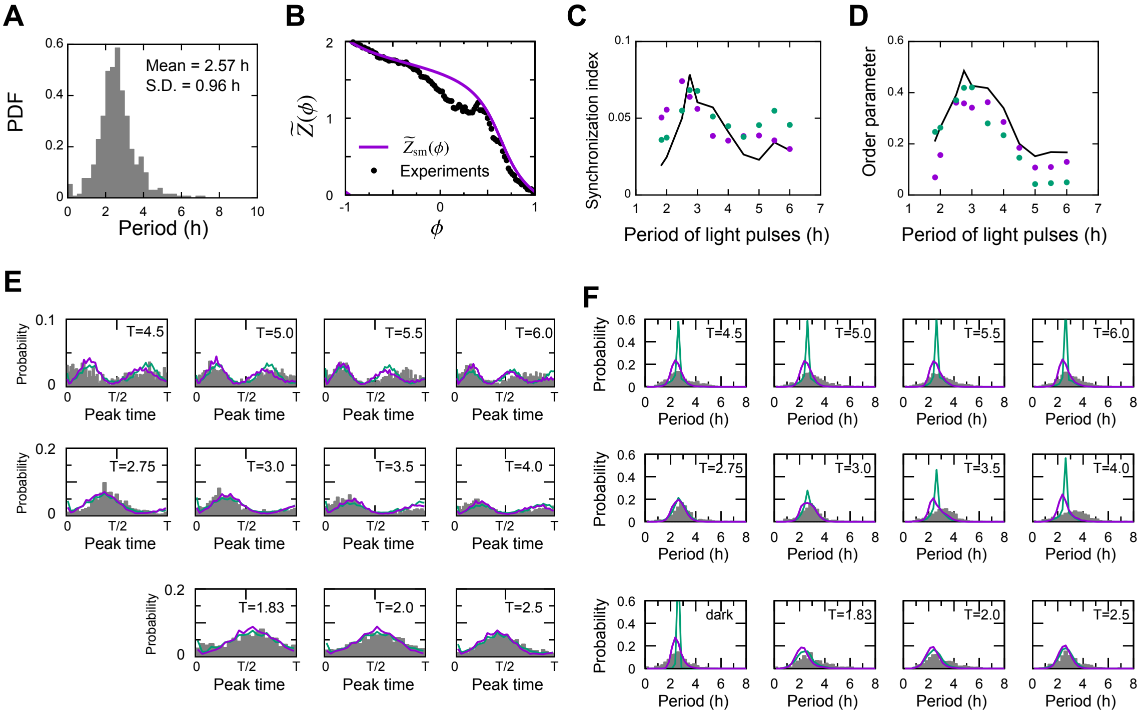

Supplementary Figure 6. Synchronization properties provided with a noisy phase model, Related to Figure 4D.

(A) Natural period distribution of *Hes1* dynamics under a dark condition observed in the experiments (gray boxes). (B) Fitting the experimentally obtained single-cell phase response curve (PRC) by using a smooth function $\widetilde{Z}_{\mathrm{sm}}(\phi)$ given by Eq.(7). The experimental PRC (black dots) was constructed from the phase-shift events in light conditions with a 2.75-h period by computing moving average. For the fitting parameters, see Supplementary Information for Numerical Simulations. (C, D) The direct estimation of PRC from experimental observations (black lines) is well fitted (purple). Synchronization index ρ (C) and Kuramoto order parameter R_1 (D) are satisfactorily reproduced using a phase model with different types of noises. Both a smooth function $\widetilde{Z}_{\mathrm{sm}}(\phi)$ (purple) and a linear interpolation function $\widetilde{Z}_{\mathrm{li}}(\phi)$ (green) succeeded in recapitulating experimental results. (E,F) Peak time and period distributions. Peak-time distributions (E) and period distributions (F) are shown. The phase model given by Eq.(4) reproduced the experimental results of the phase information (gray) by using the modeled PRCs, $\widetilde{Z}_{\mathrm{sm}}(\phi)$ (purple) and $\widetilde{Z}_{\mathrm{li}}(\phi)$ (green).

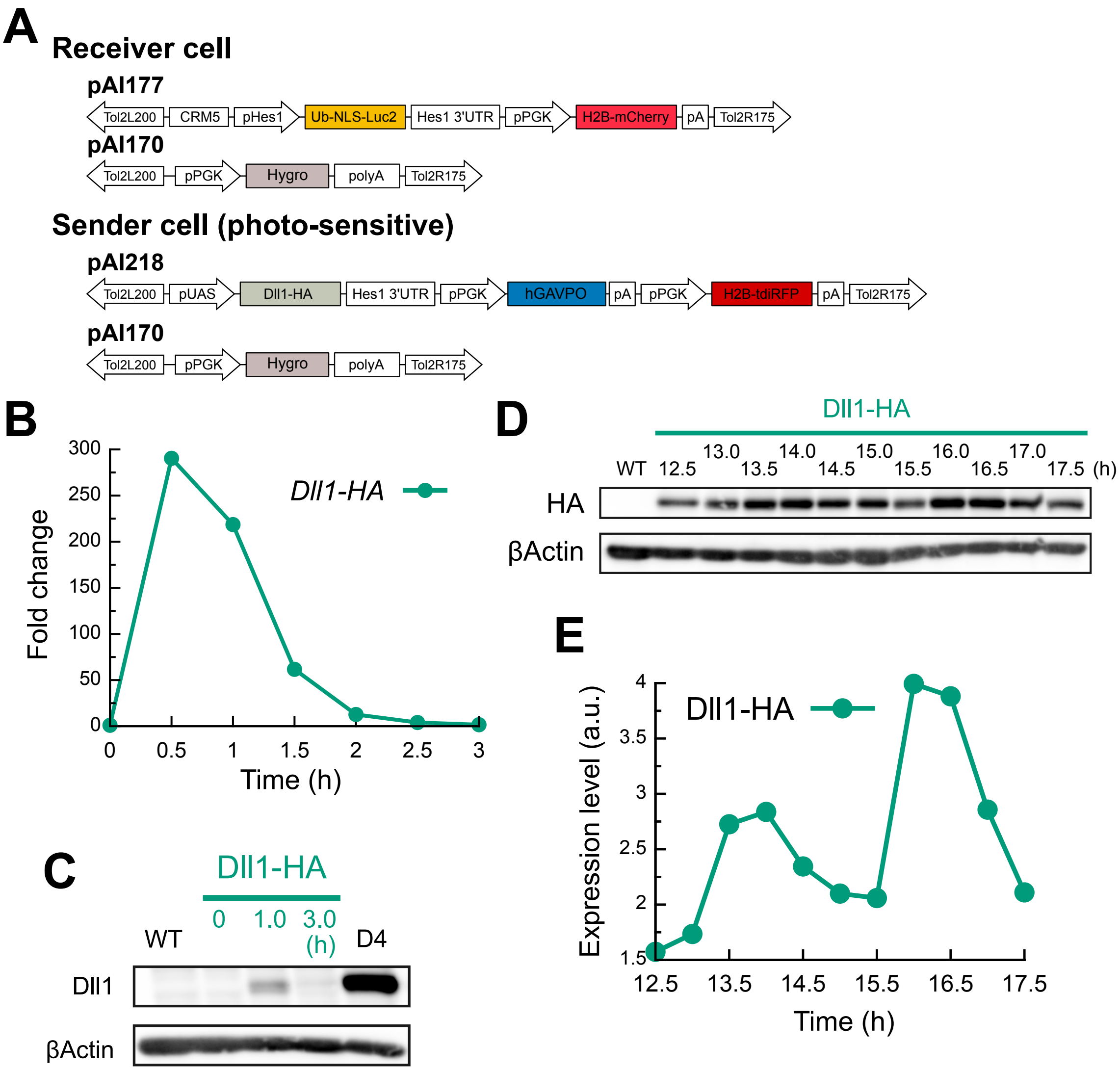

Supplementary Figure 7. Photo-inducible sender cells enables temporal control of Dll1 protein expression, Related to Figure 5. (A) Schematic structures of the plasmid constructs used for the photo-insensitive receiver cells and photo-sensitive sender cells. (B) Temporal patterns of light-induced *Dll1-HA* mRNA expression levels quantified by real-time PCR analysis. (C) Western blotting analysis shows that the expression level of Dll1 proteins induced by single light illumination (Dll1-HA, 1.0 h) is much higher than the endogenous level (WT), but lower than the ones of feeder cells (D4). See also Figs. S9C and S9D. (D,E) Western blotting analysis shows that oscillatory expression patterns of Dll1 proteins were induced by periodic light illumination with a 2.5-h period. In panels (B) to (E), light stimuli were applied with 2 min duration and the intensity of 24.7 W/m^2.

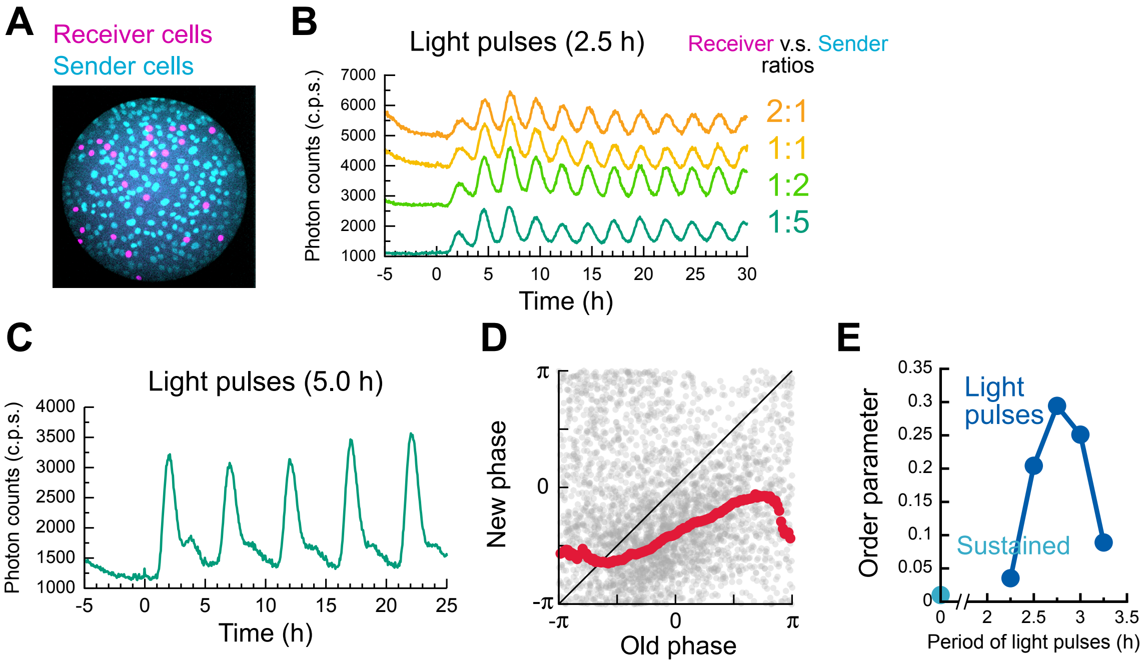

Supplementary Figure 8. Dynamic sender-receiver assay reveals the transfer of oscillatory information, Related to Figure 5. (A) A fluorescence image showing a mixed culture system of reporter oscillator cells labeled with mCherry (magenta) and photo-sensitive sender cells labeled with iRFP713 (cyan) with 1:5 mixing ratio. (B) Temporal patterns of receiver responses are robust across a variety of mixing ratios. Total cell numbers were fixed to $1.5{\times}10^5$ cells, but the ratios between the receivers and the senders were distributed from 1:5 to 2:1. (C) Population signaling-traces of Hes1 promoter activity in the presence of Dll1 perturbation with a 5.0-h period and 1-min duration light stimulus. The data in (B) and (C) were acquired by a photo-multiplier tube. (D) A single-cell phase transition curve. (E) The Kuramoto-order parameter.

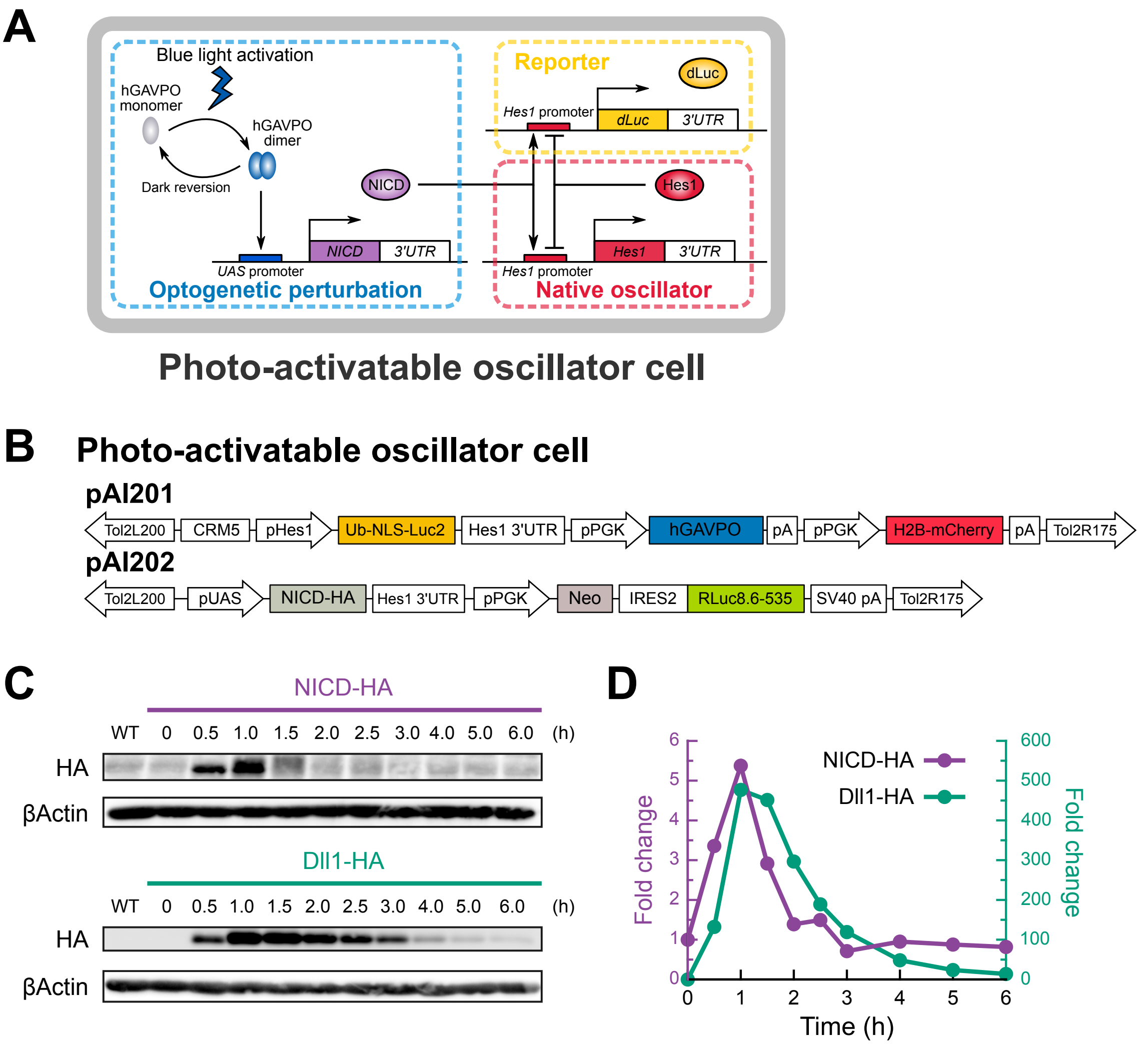

Supplementary Figure 9. Node-specific optogenetic perturbation reveals time required for ligand-receptor interactions, Related to Figure 6. (A) A schematic diagram of photo-activatable ocsillator cells for the node-specific perturbation in Delta-Notch signaling pathway. (B) Schematic structures of the plasmid constructs used for generating the photo-activatable oscillator cells. (C,D) Western blotting analysis of time-courses of NICD-HA and Dll1-HA proteins induced by single light illumination with 2 min duration and the intensity of 24.7 W/m^2.

Plasmid name	Genes	Descriptions
pCAGmT2TP	mT2TP	Expression vector for Tol2 transposase (Yagita et al., 2010)
pAI201	dLuc, hGAVPO, mCherry	*Hes1* reporter with photo-responsive module and tracking marker
pAI189	Hes1, Neo, RLuc8.6-535	
pAI202	NICD, Neo, RLuc8.6-535	Mouse Notch1 (1765-2531) fragment was used for construction
pAI235	Dll1, tdiRFP, Hygro	*Dll1* expression is driven by constitutive promoter, pPGK
pAI177	dLuc, mCherry	*Hes1* reporter with tracking marker
pAI170	Hygro	Selection for transfected cells
pAI218	Dll1, hGAVPO, tdiRFP	
pAI207	dLuc, hGAVPO, mCherry	*Hes1* 3'UTR
pAI212	dLuc, hGAVPO, mCherry	*IL-2* 3'UTR
pAI229	dLuc, hGAVPO, mCherry	*cFos* 3'UTR
pAI219	dLuc, hGAVPO, mCherry	SV40 polyA

Supplementary Table 1. The characteristics of the plasmids used in this study

Cell line	Plasmids	Figures	Descriptions
207CL2	pAI207, pAI170	Figs. 1 and S1	A photo-inducible dLuc reporter cell line of clone 2. Hygro resistant.
IL2	pAI212, pAI170	Fig. 1	A photo-inducible dLuc reporter cell line. Hygro resistant.
cFos	pAI229, pAI170	Fig. 1	A photo-inducible dLuc reporter cell line. Hygro resistant.
SV40	pAI219, pAI170	Fig. S1	A photo-inducible dLuc reporter cell line. Hygro resistant.
1A3	pAI201, pAI189	Figs. 2-4 and S2-5	A photo-sensitive oscillator cell line of clone 3. Neo resistant.
D4	pAI235	Figs. 2-4 and S2-5	A Dll1 expressing feeder cell line of clone 4. Hygro resistant.
CRM5	pAI177, pAI170	Figs. 5, 6 and S7	*Hes1* reporter (photo-insensitive) cell line of clone 5. Hygro resistant.
D1S2	pAI218, pAI170	Figs.5, 6 and S7	A photo-inducible Dll1 sender cell line of clone 2. Hygro resistant.
1C	pAI201, pAI202	Fig. S9	A photo-activatable oscillator cell line. Neo resistant.

Supplementary Table 2. List of cell lines used in this study

Movie S1. Synthetic genetic oscillations induced by light

Time-lapse movie of photo-sensitive reporter cells under the control of 3.0 h period blue light illumination (related to Fig.1).

Movie S2. Free-running oscillations of photo-sensitive oscillator cells under dark conditions

Movie S3. Optogenetic perturbation of photo-sensitive oscillators by light pulses

These time-lapse movies show gene expression dynamics of photo-sensitive oscillator cells in the absence (movie S2) or the presence (movie S3) of blue-light illumination with 2.75 h period. Left field-of-views represent luminescence images, whereas right field-of-views display colored phase maps (See also Fig. S4E).

Movie S4. Dynamic sender-receiver assay by sustained light illumination

Movie S5. Dynamic sender-receiver assay by periodic light illumination

These time-lapse movies show gene expression dynamics of photo-insensitive reporter cells co-cultured with photo-sensitive sender cells (related to Figs. 5 and S8). Sustained (movie S4) or 2.75-h period (movie S5) blue-light illumination were applied to the co-cultures. Left field-of-views represent luminescence images, whereas right field-of-views display colored phase maps.

In movies S1 to S5, blue rectangles on the upper-right side indicate timings of light illumination and white numbers denote elapsed time after starting light illumination.

References

Carr, A.-J. F. and Whitmore, D. (2005). Imaging of single light-responsive clock cells reveals fluctuating free-running periods. Nat. Cell Biol. *7*, 319–321.

Downs, T. D. and Mardia, K. V. (2002). Circular regression. Biometrika *89*, 683–698.

Eldar, A. and Elowitz, M. B. (2010). Functional roles for noise in genetic circuits. Nature *467*, 167–173.

Filonov, G. S., Piatkevich, K. D., Ting, L.-M., Zhang, J., Kim, K. and Verkhusha, V. V. (2011). Bright and stable near-infrared fluorescent protein for in vivo imaging. Nat. Biotechnol. *29*, 757–761.

Granada, A., Hennig, R., Ronacher, B., Kramer, A. and Herzel, H. (2009). Phase response curves: elucidating the dynamics of coupled oscillators. Methods in Enzymology *454*, 1–27.

Imayoshi, I., Isomura, A., Harima, Y., Kawaguchi, K., Kori, H., Miyachi, H., Fujiwara, T., Ishidate, F. and Kageyama, R. (2013). Oscillatory control of factors determining multipotency and fate in mouse neural progenitors. Science *342*, 1203–1208.

Jeziorska, D. M., Koentges, G. and Vance, K. W. (2012). Novel *cis*-regulatory modules control expression of the hairy and enhancer of split-1 (HES1) transcription factor in myoblasts. J. Biol. Chem. *287*, 5687–5697.

Kawakami, K. (2007). Tol2: a versatile gene transfer vector in vertebrates. Genome Biol. *8*, S7.

Kellogg, R. and Tay, S. (2015). Noise facilitates transcriptional control under dynamic inputs. Cell *160*, 381–392.

Kiss, I. Z., Rusin, C. G., Kori, H. and Hudson, J. L. (2007). Engineering complex dynamical structures: sequential patterns and desynchronization. Science *316*, 1886–1889.

Kobayashi, T., Mizuno, H., Imayoshi, I., Furusawa, C., Shirahige, K. and Kageyama, R. (2009). The cyclic gene Hes1 contributes to diverse differentiation responses of embryonic stem cells. Genes Dev. *23*, 1870–1875.

Kuramoto, Y. (1984). Chemical Oscillations, Waves, and Turbulence. Dover, New York.

Lin, Y., Li, Y., Crosson, S., Dinner, A. R. and Scherer, N. F. (2012). Phase resetting reveals network dynamics underlying a bacterial cell cycle. PLoS Comput. Biol. *8*, e1002778–.

Loening, A. M., Wu, A. M. and Gambhir, S. S. (2007). Red-shifted Renilla reniformis luciferase variants for imaging in living subjects. Nat. Meth. *4*, 641–643.

Masamizu, Y., Ohtsuka, T., Takashima, Y., Nagahara, H., Takenaka, Y., Yoshikawa, K., Okamura, H. and Kageyama, R. (2006). Real-time imaging of the somite segmentation clock: revelation of unstable oscillators in the individual presomitic mesoderm cells. Proc. Natl Acad. Sci. USA *103*, 1313–1318.

Mondragon-Palomino, O., Danino, T., Selimkhanov, J., Tsimring, L. and Hasty, J. (2011). Entrainment of a population of synthetic genetic oscillators. Science *333*, 1315–1319.

Pikovsky, A., Rosenblum, M. and Kurths, J. (2001). Synchronization. Cambridge University Press.

Tass, P., Rosenblum, M. G., Weule, J., Kurths, J., Pikovsky, A., Volkmann, J., Schnitzler, A. and Freund, H.-J. (1998). Detection of $n : m$ phase locking from noisy data: application to magnetoencephalography. Phys. Rev. Lett. *81*, 3291–3294.

Teng, S.-W., Mukherji, S., Moffitt, J. R., de Buyl, S. and O'Shea, E. K. (2013). Robust circadian oscillations in growing Cyanobacteria require transcriptional feedback. Science *340*, 737–740.

Winfree, A. T. (2001). The Geometry of Biological Time. Springer-Verlag, New York.

Yagita, K., Yamanaka, I., Emoto, N., Kawakami, K. and Shimada, S. (2010). Real-time monitoring of circadian clock oscillations in primary cultures of mammalian cells using Tol2 transposon-mediated gene transfer strategy. BMC Biotechnology *10*, 3.

Yamaguchi, Y., Suzuki, T., Mizoro, Y., Kori, H., Okada, K., Chen, Y., Fustin, J.-M., Yamazaki, F., Mizuguchi, N., Zhang, J., Dong, X., Tsujimoto, G., Okuno, Y., Doi, M. and Okamura, H. (2013). Mice genetically deficient in Vasopressin V1a and V1b receptors are resistant to jet lag. Science *342*, 85–90.
